# *Isatis tinctoria* L. (Woad): A Review of Its Botany, Ethnobotanical Uses, Phytochemistry, Biological Activities, and Biotechnological Studies

**DOI:** 10.3390/plants9030298

**Published:** 2020-03-01

**Authors:** Jasmine Speranza, Natalizia Miceli, Maria Fernanda Taviano, Salvatore Ragusa, Inga Kwiecień, Agnieszka Szopa, Halina Ekiert

**Affiliations:** 1 Foundation “Prof. Antonio Imbesi”, University of Messina, Piazza Pugliatti 1, 98122 Messina, Italy; j.speranza93@gmail.com; 2 Department of Chemical, Biological, Pharmaceutical and Environmental Sciences, University of Messina, Viale Palatucci, 98168 Messina, Italy; mtaviano@unime.it; 3 Department of Health Sciences, University ‘Magna Graecia’ of Catanzaro, V. Europa, IT-88100 Catanzaro, Italy; sragusa@unicz.it; 4 Chair and Department of Pharmaceutical Botany, Jagiellonian University, Medical College, Medyczna 9, 30-688 Kraków, Poland; inga.kwiecien@uj.edu.pl (I.K.); a.szopa@uj.edu.pl (A.S.); mfekiert@cyf-kr.edu.pl (H.E.)

**Keywords:** alkaloids and flavonoids, anti-inflammatory, anti-tumour, antiviral, antioxidant, Brassicaceae, hairy roots, woad

## Abstract

*Isatis tinctoria* L. (Brassicaceae), which is commonly known as woad, is a species with an ancient and well-documented history as an indigo dye and medicinal plant. Currently, *I. tinctoria* is utilized more often as medicinal remedy and also as a cosmetic ingredient. In 2011, *I. tinctoria* root was accepted in the official European phytotherapy by introducing its monograph in the European Pharmacopoeia. The biological properties of raw material have been known from Traditional Chinese Medicine (TCM). Over recent decades, *I. tinctoria* has been investigated both from a phytochemical and a biological point of view. The modern in vitro and in vivo scientific studies proved anti-inflammatory, anti-tumour, antimicrobial, antiviral, analgesic, and antioxidant activities. The phytochemical composition of *I. tinctoria* has been thoroughly investigated and the plant was proven to contain many valuable biologically active compounds, including several alkaloids, among which tryptanthrin, indirubin, indolinone, phenolic compounds, and polysaccharides as well as glucosinolates, carotenoids, volatile constituents, and fatty acids. This article provides a general botanical and ethnobotanical overview that summarizes the up-to-date knowledge on the phytochemistry and biological properties of this valuable plant in order to support its therapeutic potential. Moreover, the biotechnological studies on *I. tinctoria*, which mainly focused on hairy root cultures for the enhanced production of flavonoids and alkaloids as well as on the establishment of shoot cultures and micropropagation protocols, were reviewed. They provide input for future research prospects.

## 1. Introduction

The genus *Isatis* L., which belongs to the Brassicaceae family, comprises about 80 herbaceous annual to perennial species diffused in the Middle East and central Asia and extending to the Mediterranean region [1,2,3].

*Isatis tinctoria* L., which is commonly known as woad, is an herbaceous biennial or short-lived perennial species [1,2]. This species is thought to be native of Central Asia, as confirmed by genetic analyses [4,5], even though other authors report it as native to South-eastern Russia to South-west Asia and, perhaps, to some parts of South-eastern Europe [2,6]. 

Currently, it is a common ruderal plant of dry and sunny locations and it grows spontaneously in the rocky substrates, abandoned crops, open woods, clearings, and along the roads. It is very widespread from the sea level up to the maximum altitude of 1900 meters and it usually prefers hot spots and nitrogen-rich limestone soils [7,8]. It is considered a noxious weed in most of the Western United States [9].

*I. tinctoria* has a long and well-documented history for its medicinal properties and its indigo blue colour. This double use of woad is shown by its name. On one hand, the term “*Isatis*” that derives from the latin word “Isazein” and the greek word “Isadso” is linked to the its ancient use to treat wounds [10]. On the other hand, the term “*tinctoria*” refers to the use that was done in the dye works [8].

This species was introduced in Europe in late Prehistory and Protohistory periods, as confirmed by the discovery of textile remains dyed in blue and preserved from the Neolithic, Bronze, and Iron Ages [11]. The ancient Egyptians used *I. tinctoria* as an indigo source to dye the cloth wrappings applied for the mummies [12].

Historical accounts about the use of indigo in Europe date back to Roman times. Historical sources report that Celtic and Germanic people used woad to paint their body and hair for prophylactic or ritual purposes. 

Pliny the Elder often mentioned woad in its writings, and reported the custom of female Britons covering their bodies with indigo blue for religious ceremonies [13]. Julius Caesar reported in his book *De Bello Gallico* that the Celtic populations used woad indigo to colour themselves to generate a fearsome appearance [14]. They pricked their skin and rubbed woad on to form a blue tattoo. The Romans called these people *Picti*, which means “painted people” [13]. This suggests that the *I. tinctoria* dye may have been used both for textile and body art [11].

From the 12th up to the 17th century, *I. tinctoria* has been widely cultivated in Europe (Germany, France, England, and Italy), and extensively used as indigo dye and medicinal plant. In the early 17th century, *I. tinctoria* was intentionally taken from Europe into North America by early colonists as a textile dye crop [6,15]. In the late 17th century, the decline of the woad industry in Europe was initiated due to the import of indigo blue from *Indigofera tinctoria* cultivated in Asia (India, Bangladesh) and, afterward, from other *Indigofera* species in the Caribbean and the American colonies, which was easier and more economical to extract. The crop was definitively abandoned in the late 19th century, when the production of synthetic dyes completely replaced natural indigo production [5,14,16]. With the declining importance as a dye and the disappearance of woad cultures, the plant also fell into oblivion as a medicinal plant [6,17]. 

Currently, *I. tinctoria* is widely utilized for medicinal purposes in the Traditional Chinese Medicine (TCM) [6,18,19] and, from the 2011 year, it is recognized as a pharmacopoeial plant in Europe [20].

Currently, *I. tinctoria* is utilized in cosmetic industries for the production of soaps and body creams. The seed oil and the leaves (powder/extract) are cosmetic ingredients for skin and hair conditioning due to their emollient and moisturising properties [5]. The roots (powder/extract) have astringent and skin protecting properties. The CosIng Database elaborated by the European Commission gives positivity to these previously mentioned raw materials for the production of cosmetics in Europe [21]. 

In recent years, renewed interest in natural dyes showed by the dyestuff industry and its potential use for medicinal and cosmetic products has encouraged growers to reintroduce *I. tinctoria* crops in the European agricultural system [5,22]. Horticulturists have become interested in utilizing *I. tinctoria* as an ornamental plant because of its tolerance to heat and water stresses, a long flowering period, and attractive inflorescences [10].

Currently, although this species is not considered an edible vegetable worldwide, rural people living in Sicily (Italy) around Vulcan Etna consume boiled flower buds of this plant as ingredients for salads and omelettes [10,23]. 

Due to the relevance of *I. tinctoria*, in recent decades, a considerable number of phytochemical and biological investigations on this species have been carried out. A previous review article by Hamburger [6] reported the studies published from 2000 to 2002 on the anti-inflammatory activity of *I. tinctoria* extract and its active component tryptanthrin. 

The aim of this article is to provide an up-to-date and comprehensive overview of the ethnobotany, phytochemistry, and the biological properties demonstrated for this valuable species in order to support its therapeutic potential and to provide input for future research prospects. The article also focuses on all attempts in plant biotechnology studies for the enhanced production of flavonoids and alkaloids from hairy root cultures as an alternative to plant raw materials, but also on the early result about the establishment of shoot cultures and micropropagation protocols.

## 2. Botanical Description

**Figure 1:**
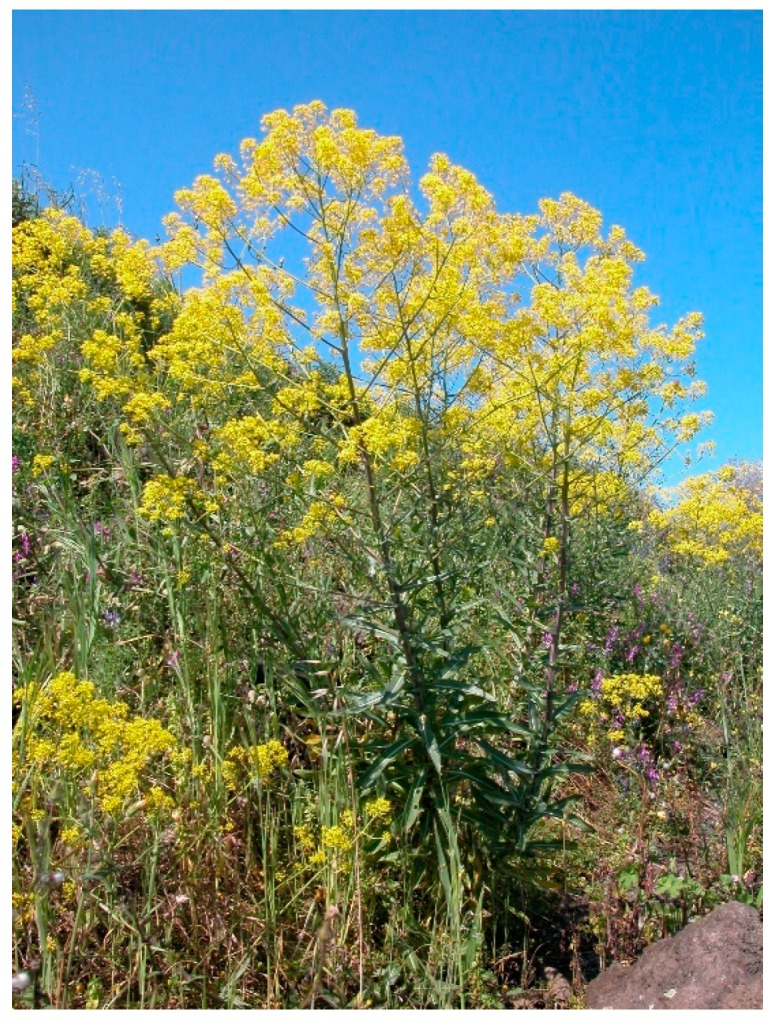
*Isatis tinctoria* L.

*Isatis tinctoria* L. (woad) is an herbaceous biennial or short-lived perennial species, more or less hairless to hairy, greyish with an erect stem, up to 120 cm in height, simple below, and branched above (Figure 1). 

The root is cylindrical, slightly tortuous, externally greyish-yellow, or brownish-yellow, wrinkled longitudinally and lenticellate transversally, with rootlets or rootlet scars. Root stock slightly expanded, which exhibited dark green or dark brown petiole bases arranged in whorls, and dense tubercles. Basal leaves are oblong-lanceolate, entire to toothed, and long-petioled. Cauline leaves, which are narrower than basal and gradually reduced upwards, are simple, entire, sagittate, usually amplexicaul, with acute auricles. The flowers are gathered in a racemose inflorescence, with yellow petals, tetradynamous androecium, consisting of six stamens with two filaments shorter than the others. Fruits are pendulous siliques, oblong-obovate, or elliptic-obovate hairless or shortly hairy. This species is very variable, particularly in the size, shape, and hairiness of the silique [1,2,13,24]. 

There are conflicting views on the taxonomy of *I. tinctoria* (European woad) and *I. indigotica* (Chinese woad). The latter was described for the first time by Fortune in 1846, and initially considered as a distinct species. Afterward, some taxonomists have classified it as a variety of *I. tinctoria* [17]. Angelini et al. [25] reported that *I. indigotica* presents morphological, genetic, and physiological differences with respect to the European *I. tinctoria*, even though it is closely related to this one. The seedlings of both species develop a rosette in the first year of their cycle. The leaves *I. indigotica* show a glaucous instead of a shiny surface, which is rarely pubescent with a greater thickness and a more upright habit. The high degree of genetic diversity between the two species was confirmed by various studies [26,27]. Despite this, *I. indigotica* is currently considered a synonym of *I. tinctoria*, and not a separate species, as confirmed by the consulted taxonomic databases known as The Plant List, International Plant Names Index (IPNI), and Tropicos. On the other hand, the Euro+Med PlantBase does not mention *I. indigotica* in the list of synonyms.

## 3. Ethnobotanical Uses

*I. tinctoria* has a long and well documented history as a medicinal plant in both Eastern and Western cultures. Its therapeutic properties have been esteemed in Europe and in Traditional Chinese Medicine (TCM) for centuries. 

The first written records in Europe about the medicinal uses of this plant were by Hippocrates (460 b.C.) who supported its use for treating wounds, ulcers, and haemorrhoids. Galen (129–216 b.C.) and Pliny (23–79 b.C.) recommended it as well [6,28]. 

Due to the huge cultivation in Europe for the production of indigo dye, from the 12th century until the 17th century, *I. tinctoria* was also extensively used as a medicinal plant to treat snake bites, wounds, and other inflammatory ailments [6,29]. These medicinal properties have been widely described in a number of Renaissance and Baroque herbal texts, which recommended woad for treating haemorrhoids, ulcers, and tumours [6]. 

Various authors have mentioned different medicinal uses for *I. tinctoria* leaves and roots. The leaves have been utilized in the treatment of typhoid, measles, and the flu [30]. Garland [31] reported their use against the tremendous inflammation of the skin due to St. Anthony’s fire, which is a dreaded illness that was common in the Middle Ages. Other uses such as treating iposideremic anaemic diseases (due to iron deficiency), scurvy, and, in general, hypovitaminosis have been correlated to the relevant levels of iron, and of vitamins A and C present in the leaves. Moreover, the leaf extracts have been used for their anabolic, astringent, and detergent properties. According to these skills, the extracts were utilized in treating acrodermatitis, haemorrhagic diathesis, eczema from scrofulous, furunculosis, intoxications, hypercholia, jaundice, gastric neurosis, hives, torpid sores, scrofulous, heartburn, and seborrhoea [32]. 

The bitter and refreshing roots have been utilized to reduce scarlet fever. The root extracts have been used to treat patients with solid tumours and leukaemia, which is a traditional usage that led to purification of indirubin [9].

After *I. tinctoria* lost its importance as a source of indigo for dying, its curative properties in Europe fell into oblivion. It has only just recently emerged into Western awareness as having medicinal properties, which is mostly due to its antiviral properties [28]. The European Pharmacopoeia currently reports the monograph of *I. tinctoria* root (Isatidis radix) for use in official European phytotherapy [24].

In China, woad has an equally rich history as dye and a medicinal plant. The Chinese Pharmacopoeia reports three monographs for *I. tinctoria*: *Băn Lán Gēn* (Isatidis radix), *Dà Qīng Yè* (Isatidis folium), and *Qing Dai* (Indigo naturalis) [9,33]. These herbal preparations are considered as somewhat differing medicines.

*Băn Lán Gēn* (Isatidis radix) is the dried root of *I. tinctoria* harvested in some provinces of R.P. China such as Hebei, Jiangsu, and Anhui during the autumn, dried in the sun, and processed into granules (Banlangen Keli). The product is very popular throughout China and it is most commonly consumed when dissolved in hot water or tea [34]. It reduces the body temperature and soothes the sore throat. It is indicated for eruptive epidemic diseases, pharyngitis, laryngitis, scarlet fever, erysipelas, and carbuncles. It is utilized for treating hepatitis, mumps, the flu, mononucleosis, viral skin diseases such as herpes simplex, herpes zoster, and pityriasis rosea, epidemic cerebrospinal meningitis, and diphtheria [34,35,36,37]. *Băn Lán Gēn* has been one of the eight major medicines recommended by the Chinese government for preventing and controlling the deadly severe acute respiratory syndrome (SARS) [38]. 

*Dà Qīng Yè* (Isatidis folium) consists of *I. tinctoria* dried leaves [39] collected in the summer and the autumn from different provinces of R.P. China such as Hebei, Jiangsu, Anhui, and Henan and dried in the sun. *Dà Qīng Yè* is used against the virus of B-encephalitis, mumps, and influenza and for treating the leptospirosis [36].

*Qing Dai* (Indigo naturalis) is a dark blue powder prepared from the leaves of various plants including *I. tinctoria*, *Baphicacanthus cusia* (Nees) Bremek., *Indigofera suffruticosa* Mill., *Indigofera tinctoria* L., or *Polygonum tinctorium* Ait. It is used in TCM as a haemostatic, antipyretic, anti-inflammatory, and in the treatment of bacterial and viral infections [40,41].

Another preparation, called *Dang Gui Long Hui Wan*, consisting of 11 herbal medicines including *I. tinctoria*, has been used in TCM as a remedy for various chronic diseases including chronic myelocytic leukaemia [14]. 

Clinical trials have not assessed the safety of *I. tinctoria* leaf or root preparations. Traditional Chinese herbal texts do not list adverse effects, except gastro-intestinal disorders such as nausea and vomiting in some patients [36,42].

## 4. Chemical Composition

*I. tinctoria*, like other species belonging to the Brassicaceae family, shows an interesting chemical profile characterized by a large variety of compounds. *I. tinctoria* represents a valuable source of bioactive compounds such as alkaloids, phenolic compounds, polysaccharides, glucosinolates, carotenoids, volatile constituents, and fatty acids (Table 1).

### 4.1. Alkaloids

**Figure 2:**
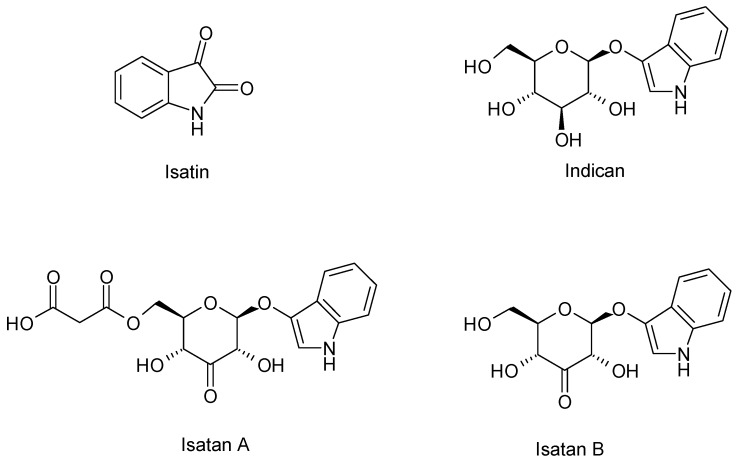
Chemical structure of indigo precursors.

*I. tinctoria* is an important source of two known indolic alkaloids called indigo and indirubin. The first one has a blue colour instead of the second one that has a red colour and both of them are extensively used to dye textiles, cosmetics, foods, and pharmaceutical preparations. The plant is not able to synthetize directly into the indigoid pigments, but it produces several precursors: indican, isatan A, isatan B, and isatan C (Figure 2). When the leaves are damaged and exposed to air, precursors are submitted to enzymatic hydrolysis by β-d-glucosidase and β-glucuronidase. After cleavage of the O-glycosidic linkage, the indoxyl is released giving, in turn, indigo (blue indigoid pigments) through the oxidation process, and producing isatin as a side reaction thanks to an oxygen-rich environment. Eventually, the condensation of indoxyl with isatin produces indirubin (red indigoid pigment), whereas the condensation with dioxindole, coming from Isatan C, produces isoindirubin (red indigoid pigment) [44].

The indigo precursors indicant as well as isatans B and C were detected by Maugard et al. [44] in the acetone/acetic acid 1% *v*/*v* extracts of *I. tinctoria* young and old fresh leaves. Moreover, the number of indigo precursors in these samples was quantified by the same team, who established that isatan B was the major precursor. The concentration of indigo precursors was higher in the young, fresh leaves than in the oldest ones.

A later study of Oberthur et al. [53]. about *I. tinctoria* liophylized or air-dried leaves identified isatan A as the major indoxyl glycoside and gave the correct structure of isatan B, which was previously assigned by Epstein et al. [54]. Furthermore, the same team showed how the content of indigo precursors strictly depended on the post-harvest treatments. A mix of *I. tinctoria* young and older rosette leaves were submitted to three different post–harvest treatments: immediate shock freezing with liquid N_2_ followed by freeze drying, air drying at room temperature, and drying in a thermostatic oven at 40 °C. The highest concentrations of isatans A and B were found in freeze dried leaf samples whereas the indican had the lowest amount. On the other hand, the other two treatments lead to high concentrations of indican and complete disappearance of isatans A and B. In all samples, the isatan B concentrations were lower than those of isatan A. These data and the enzymatic conversion of indican to isatan B underlined the biosynthetic pathway where isatan B derived from indican is, at the same time, a biogenetic precursor and degradation product of isatan A. Eventually, the production and amount of indigo precursors depend on the cultivars and harvest period, which also influenced the indigo dyes’ composition [55].

**Figure 3:**
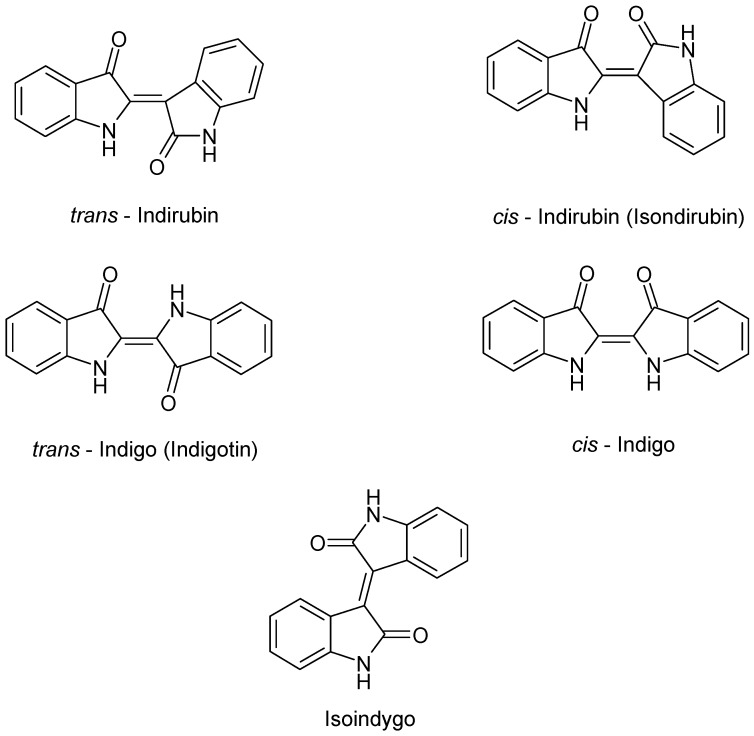
Chemical structure of indigo dyes.

In addition to the indigo precursors, indigoid pigments named *trans*-indigo (indigotin, blue), *trans*-indirubin (isoindigotin, red), *cis*-indirubin (isoindirubin, red), iso-indigo (indigo brown), and *cis*-indigo were detected by HPLC in the acetone/acetic acid 1% *v*/*v* extracts of *I. tinctoria* young and old fresh leaves (Figure 3) [44]. It is also important to point out that the indigo dyes indigo and indirubin appear under the fermentative conditions, used in the old natural indigo production, or after a drying process at 40 °C [45].

Beyond the study of *I. tinctoria* fresh leaves, one unique analysis on dried rosette leaf extracts were carried out by Mohn et al. [33]. Thanks to the extractions performed through two different solvents, known as dichloromethane and methanol, it was possible to obtain a broad-based characterisation of the *I. tinctoria* profile. The LC-MS analysis of dichloromethane extracts showed various indolic alkaloids such as isatin, isoindigo, indoxyl indigo, and indirubin. Instead, in the methanolic extracts, only indican was detected [33].

Recently, LC-MS analysis of *I. tinctoria* frozen and lyophilized and dried leaf extracts were performed and the obtained metabolite profiles were compared. In the lyophilized extracts’ analysis, beyond the characterization and quantification of 122 compounds previously described, the following indole derivatives were described for the first time: acetylindican, malonylindican, two dioxindole glucosides, dioxindole malonylglucoside (Isatan C), 6-hydroxyindole-3-carboxylic acid 6-O glucoside, and 6-hydroxyindole-3-carboxylic acid glucose ester [45].

Several research studies carried out on *I. tinctoria* dried roots showed two new and five known indole alkaloid glycosides. The structure of the two new indole alkaloid glycosides isatindigoside A and isatindigoside B were determined by extensive spectroscopic data analysis including 1 D, 2 D NMR, IR, and HR-ESI-MS data analysis. The known glycosides isatindosulfonicacid A 3-O-b-d-glucopyranoside, indole-3-acetonitrile 6-O-b-d-glucopyranoside, isatindigobisindoloside A, isatindigobisindoloside B, and isatindigobisindoloside F, were identified by comparing spectroscopic and optical rotation data [37]. Furthermore, a new indole alkaloid with an unusual carbon skeleton named isatisindigoticanine A were detected by the same team [56].

**Figure 4:**
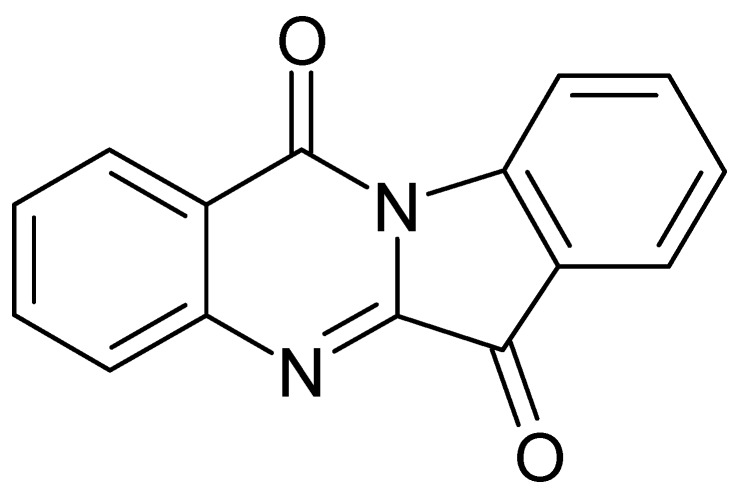
Chemical structure of the alkaloid tryptanthrin.

Another important compound of this class is tryptanthrin, indolo-[2,1-b]-quinazoline alkaloid (Figure 4), which is also responsible for some biological activities of *I. tinctoria*. Honda et al. [43] isolated and identified tryptanthrin from *I. tinctoria* dried rosette leaf chloroform extracts for the first time. Moreover, more than 70 *Isatis* samples of different origin were analysed and the tryptanthrin content in the leaves varied from 0.56 to 16.74 × 10^−3^% [57]. The tryptanthrin synthetic pathway is not known, but it is a product of the post-harvest process. Its formation is apparently favoured by the drying process and elevated temperature (40 °C) and, on the contrary, the lyophilization and fermentative conditions decreased its concentration [58].

### 4.2. Phenolic Compounds

The main class of secondary metabolites in *I. tinctoria* polar extracts is represented by phenolic compounds [16,47]. Phenolics can be divided into three main categories: flavonoids, phenolic acid, and their conjugates [16]. The identified flavonoids represent the major class of the phenolic constituent and are derivatives of flavones and of flavonols [46].

In methanolic extract from dried leaves, the presence of *p*-hydroxybenzoic, *o*-methoxybenzoic, *p*-methoxybenzoic, dihydrocaffeic, and 4-hydroxy-3-methoxyphenylpropanoic acid has been reported [48]. They found methyl esters of several phenolic acids, like ferulic, sinapic, salicylic, vanillic, and 4-hydroxyphenylacetic acids as a result of acidic hydrolysis. 

**Figure 5:**
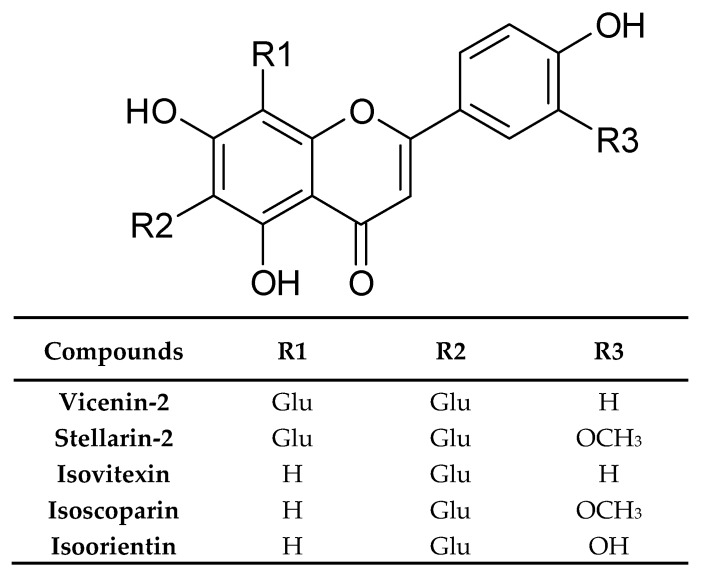
Chemical structure of representative flavonoids of *I. tinctoria* L.

The same kind of extract was analysed by Mohn et al. [33]. In dried rosette leaves, they found vicenin-2, stellarin-2, isoorientin, isovitexin, isoscoparin, and some of their glucosides (Figure 5). Simultaneously, the isocoparin, sinapic, and ferulic acids turned out from the analysis of dichloromethane extract from the same plant material. 

The drying process may cause changes in the metabolite profile even among phenolic compounds. On this account, lyophilization of freshly harvested plant material was introduced to prevent biochemical decomposition, e.g., deglycosylation. As a result of this procedure, a large group of conjugates between hydroxycinnamic acids and a hexose or dihexose (10 compounds), glycerate (two compounds), malate (one compound), or glucaric acid (45 compounds) was detected in the rosette leaf methanol extract. All of the conjugates showed one or two moieties of *p*-coumaric, ferulic, or sinapic acid. Furthermore, two glucaric acid conjugates containing a higher molecular weight dilignol-like esterified moiety in which coniferyl alcohol was linked to ferulic acid via a typical lignin 8-O-4 linkage were detected. In addition, hydroxycinnamic acids conjugated with flavone glucosides were identified. Among benzoic acid derivatives, only two compounds were confirmed, which include hexoside and hexose ester of protocatechuic acid [16].

Many flavonoids showed carbohydrate and acid moieties and, according to it, many derivatives of the flavonoid aglycones were detected. Nguyen et al. [16] was found in the polar extract from rosette leaves’ flavone compounds mono or diglicosylated on the C-6 position with a rare 1→3 glycosidic linkage. Flavone compounds with additional glycosylation of the O-7 position and esterification with a hydroxycinnamic acid derivative such as *p*-coumaric, ferulic, or sinapic acid were identified. Moreover, the same authors reported the presence of six flavone glucosides conjugated with hydroxycinnamic acid. They identified isoscoparin with its eight derivatives including: 3”-O-sinapoyl glucoside, 3”-O-feruloyl glucoside and 3”-O-*p*-coumaroyl glucosides of isoscoparin, 4′-O-feruloyl iscoscoparin-3”-O-glucoside-7-O-glucoside, 2”-O-feruloyl iscoscoparin-3”-O-glucoside-7-O-glucoside, and isoscoparin-3”-O-glucoside-7-O-feruloylglucoside. In the same extract, the luteolin-6-C-glucoside-7-O-glucoside was detected for the first time and it has not been described in other plants before.

**Figure 6:**
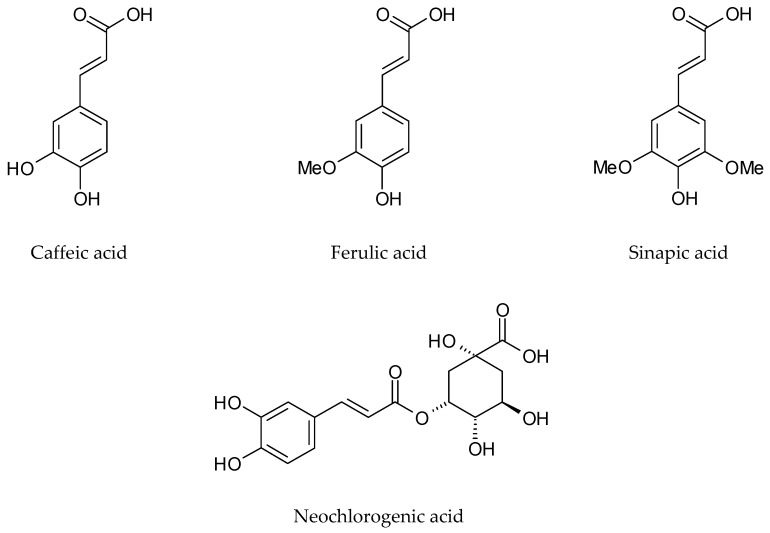
Chemical structure of representative phenolic acids of *I. tinctoria* L.

Miceli et al. [46] characterised, for the first time, the phenolic compounds of the hydroalcoholic extract (70% methanol) from *I. tinctoria* lyophilized cauline leaves. Among 13 identified flavonoids, derivatives of flavones and flavonols, which were quantitatively dominant, were vicenin-2, isovitexin, and apigenin glucosides. Some of them like kaempferol, buddleoside, and quercetin have not been detected in *Isatis* before. Moreover, the authors reported the presence of phenolic acids like neochlorogenic, chlorogenic, caffeic, ferulic, sinapic, *p*-coumaric, and coumarylquinic acids (Figure 6). Neochlorogenic and sinapic acids were dominant compounds.

The same authors also performed a comparative analysis of hydroalcoholic extracts from rosette leaves, cauline leaves, and flowers of *I. tinctoria* [47]. According to the results, the phenolic profile of both types of leaves was very similar, but cauline leaves showed more than double the phenolic content than rosette leaves. A lower number of phenolic compounds was detected in the flower extract. Nonetheless, they were found to be quantitatively close to those of cauline leaves. The main flavonoids detected in leaves and flowers from *I. tinctoria* were vicenin-2, stellarin-2, isovitexin, luteolin-glucuronide, and quercetin. Among them, vicenin-2 turned out to be the most abundant flavonoid detected in the cauline leaf extract, whereas luteolin-glucuronide and stellarin-2 were the main compounds for rosette leaves and flowers, respectively [47].

### 4.3. Glucosinolates

*I. tinctoria* is a notable source of glucosinolates, which are synthesis and storage products of isothiocyanates. When the plant tissue is damaged, glucosinolates are released and converted by myrosinase (β-thioglucosidase). This is an enzyme that coexists with these compounds in plants even though it is physically separated [19]. The enzyme hydrolyzes the glucose moiety into the main skeleton by giving isothiocyanates and, under certain conditions, this reaction may lead to other types of products like thiocyanates, indoles, epithionitriles, and nitriles [59]. These compounds are responsible for the bitter and pungent taste and for most of the biological activities of the glucosinolates such as anticancer, antioxidant, and antibacterial factors. 

Moreover, glucosinolates are known as goitrogens because they prevent the absorption of iodine, which causes swelling of the thyroid.

From the chemical point of view, glucosinolates are (Z)-N-hydroxyminosulfate esters characterized by sulphur-linked β-d-glucopyranose moiety and an amino acid-derived side chain. They are divided into three groups according to their amino acid side chain: aliphatic glucosinolates, aromatic glucosinolates, and indole glucosinolates [60].

**Figure 7:**
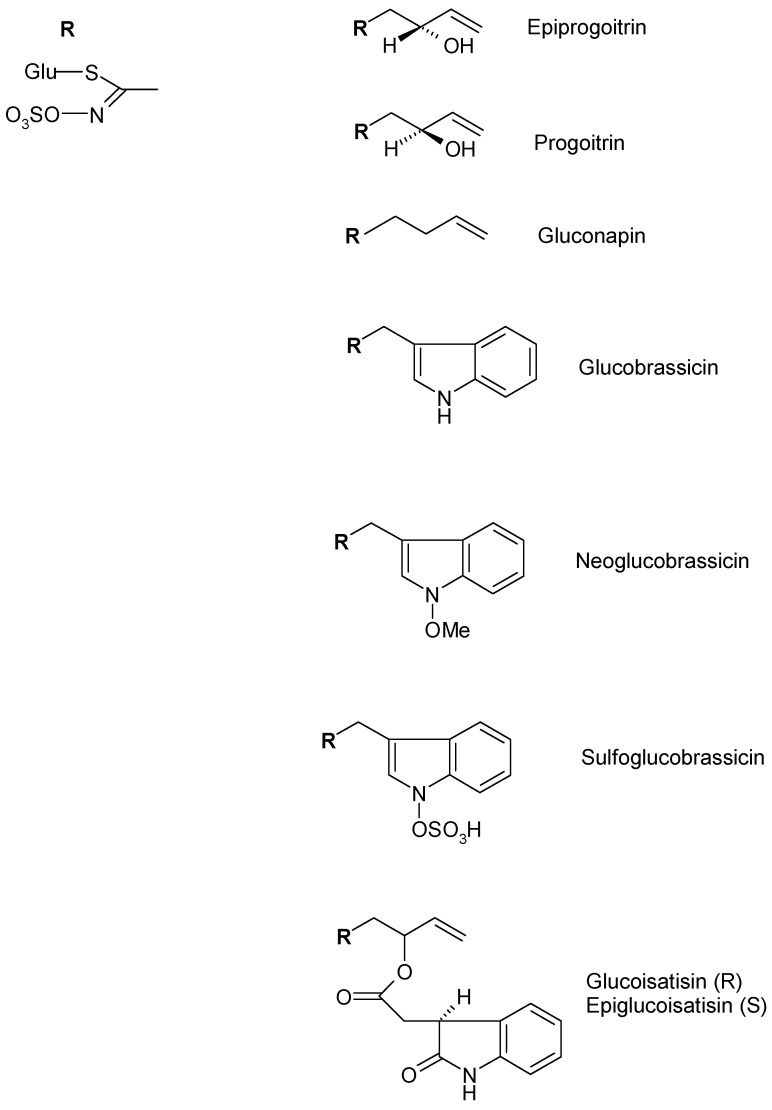
Chemical structure of representative glucosinolates of *I. tinctoria* L.

In *I. tinctoria* seeds, aliphatic glucosinolates as gluconapin, progoitrin, and epipogoitrin, which were mainly detected, but indolic glucosinolates like glucoisatisin/epiglucoisatisin (inseparable epimeric mixture present only in seeds), glucobrassicin and neoglucobrassicin were also identified. The glucosinolate pattern of the seeds extract showed differences when compared to the extract obtained from the rosette leaves. In fact, the extracts obtained from *I. tinctoria* frozen and liophylized rosette leaves, mainly contained indolic glucosinolates including glucobrassicin, neoglucobrassicin, sulfoglucobrassicin, and glucotropaeolin (Figure 7) [49,50].

The analysis carried out by the same authors also showed that the glucosinolate content in the leaf samples was higher than that of the seed samples. Taviano et al. [47] showed that a very low amount of glucoiberin, glucobrassicin, and 4-methoxyglucobrassicin are contained in the liophylized leaf (rosette and cauline) and flower of 70% methanol extracts. 

Furthermore, recent studies reported, for the first time, the presence of gluconapoleiferin in the frozen and lyophilized rosette leaf methanol extracts [16].

### 4.4. Carotenoids

Carotenoids are highly lipophilic metabolites and, for this reason, the extraction and separation conditions were optimized for the analysis. On one hand, the dichloromethane extract of *I. tinctoria* dried rosette leaves showed several known carotenoids like (all-*E*)-β-carotene and various lutein isomers [33]. On the other hand, in the hexane/acetone extract (1:1 *v*/*v*) obtained from the dried rosette leaves, it was possible to detect more and other unknown carotenoids like (all-*E*)-lutein, (*Z*)-neochrome, (15*Z*)-violaxanthin [33].

Moreover, in the same extract, the lipophilic chlorophyll degradation products phaeophytin a, b, and pyrophaeophytin a, b were identified. 

### 4.5. Monolignols and Oligolignols

Phenolic derived compounds with higher molecular weight belonging to monolignols and oligolignols were described by Nguyen et al. [16]. In methanol extracts of *I. tinctoria* frozen and lyophilized rosette leaves, monolignols and oligolignols, derived from coniferyl and synapyl alcohol, were characterized by LC-MS for the first time. Some of these compounds were previously identified in other plants such as *Arabidopsis thaliana* and *Linum usitatissimum.* Furthermore, several compounds containing hexose, guaiacyl, and syringyl units linked by 8-5 and 8-O-4 bonds were detected for the first time and all compounds were found in a hexosylated form. Authors underlined that they were accumulated in the vacuole of mesophyll and epidermal cells rather than in the cell walls of the leaf nervature. This finding suggested that oligolignols may play some physiological role in *I. tinctoria* leaves.

### 4.6. Volatile Constituents

The volatile constituents of *I. tinctoria* were characterized in leaves and roots. The first extraction from fresh leaves was carried out by Condurso et al. [19] through solid-phase microextraction and gas chromatography-mass spectrometry (SPME/GC-MS). This analysis allowed them to identify several compounds such as acids, alcohols, aldehydes, esters, ethers, furans, hydrocarbons, isothiocyanates, thiocyanates, ketones, nitriles, sulfurated compounds, monoterpenoids, and sesquiterpenoids. A high portion of volatile isothiocyanates was detected in the leaves (40% of the total volatile fraction), especially 3-butenyl isothiocyanate that is the aglicone of gluconapin. The 2-hydroxy-3-butenyl isothiocyanate, that is, the aglicone of progoitrin and epiprogoitrin, was also identified. After the isothiocyanates, aldehydes were the most represented class and both aliphatic and aromatic types were identified, especially the (*Z*)-2-esenal (a typical aldehyde from leaves). Among the sulfurated compounds, the 2-ethylthiophene was the most abundant one. Moreover, saturated and unsaturated alcohols were identified with tetradecanol as the main component. Almost 7% of the volatile fraction was represented by monoterpenoids and sesquiterpenoids. Ten monoterpenoids and two sesquiterpenoids were detected and the monocyclic monoterpenoid limonene was the major one. Furthermore, the nitriles that derive from isothiocyanates due to the loss of sulphur were detected, which were 4-pentenenitrile, 3-hydroxy-4-pentenenitrile, heptanenitrile, octanenitrile, and 2-phenylacetonitrile as the most abundant ones.

Verzera et al. [51] carried out the first extraction of *I. tinctoria* dried root volatile constituents by SPME/GC-MS and the same main classes of compounds were identified. The volatile content of roots was also characterized by a high portion of 3-butenyl isothiocyanate that represents 82% of the total volatiles. After isothiocyanates, the alcohols and nitriles were the most abundant classes and they, respectively, constituted 4.9% and 3.6% of the total volatile content. The other classes of compounds are even less represented.

### 4.7. Other Constituents

Different studies have shown that polysaccharides from roots are important bioactive components of *I. tinctoria*. In a study carried out by Han et al. [34], the conditions that gave the maximum extraction yield of the polysaccharides from *I. tinctoria* root (11.19% ± 0.04) have been established. The seeds of *I. tinctoria*, like those of other plants belonging to family Brassicaceae, show an interesting fatty acid composition, which is the highest among all *Isatis* spp. (10%). According to Kizil et al. [52], seeds contain erucic acid (26.48% of total fatty acid fraction), oleic, linoleic, linolenic acids as the major components, and other fatty acids like palmitic, stearic, arachidic, and tetracosanoic. Moreover, Mohn et al. [33] reported the presence of palmitoleic and α-lysolecithin in dichloromethane dried rosette leaf extracts. Additionally, the presence of the triterpenoid ursolic acid was confirmed by these authors.

The microelement analysis of the *I. tinctoria* seeds carried out by Kizil et al. [52] showed the presence of K (3493 mg/kg), Ca (2173 mg/kg), P (625.6 mg/kg), Mg (593.0 mg/kg), Fe (77.54 mg/kg), Al (74.78 mg/kg), Na (66.31 mg/kg), Mn (7.383 mg/kg), Ni (4.789 mg/kg), Pb (2.493 mg/kg), Cu (1.888 mg/kg), Cr (0.630 mg/kg), and Cd (0.250 mg/kg).

## 5. Biological Activities

A number of studies have been conducted to evaluate different biological activities of *I. tinctoria* in both in vitro and in vivo experimental models. Biological investigations are mainly based on the ethnopharmacological uses of the plant as an effective anti-inflammatory, anti-tumour, and antiviral herbal remedy. In addition, other biological activities such as analgesic, antimicrobial, and antioxidant have been highlighted (Table 2).

### 5.1. Anti-Inflammatory Activity

*I. tinctoria* has been appreciated for centuries in Europe and, in TCM, for its anti-inflammatory properties. For this reason, several in vivo and in vitro research studies were carried out in order to assess its anti-inflammatory potential. 

Solvents with different polarity and diverse extraction procedures (maceration, reflux extraction, CO_2_ supercritical fluid extraction (SFE), and accelerated solvent extraction (ASE)) were utilized in order to establish the best condition to extract the bioactive compounds contained in the leaves and roots of *I. tinctoria*.

One of the earliest studies investigated the effects of the treatment with an aqueous extract of *I. tinctoria* in a model of chronic *Pseudomonas aeruginosa* lung infection mimicking cystic fibrosis in rat. *I. tinctoria* (400 mg/kg b.w., s.c. once a day for 10 days) was able to decrease the frequency of the lung abscess and the severity of the macroscopic pathological changes in lungs. The *I. tinctoria* extract was also able to modulate and decrease the inflammatory response in the lungs by enhancing the shift in the inflammatory response from an acute type inflammation dominated by polymorphonuclear leukocytes to a chronic type inflammation dominated by mononuclear leukocytes [61].

The anti-inflammatory activity of lipophilic *I. tinctoria* leaf extracts was demonstrated. Recio et al. [18] showed the efficacy of SFE and dichloromethane (DCM) extracts obtained from dried rosette leaves of *I. tinctoria*, using two experimental models of acute inflammation including carrageenan-induced mouse paw oedema and 12-*O*-Tetradecanoylphorbol 13-acetate (TPA)-induced mouse ear oedema. The SFE (os, 75–125 mg/kg) and DCM (os, 125–175 mg/kg) extracts showed dose-dependent anti-inflammatory activity in the carrageenan-induced mouse paw oedema. Particularly, the potency of the SFE extract was found higher than that of DCM. In the second in vivo model of acute inflammation, the topical application of the DCM and SFE extracts (0.5 mg/ear) significantly inhibited the TPA-induced ear oedema and the topical administration was more effective than the systemic one.

In sub-chronic inflammation induced by repeated application of TPA in the mouse ear, both oral (150 mg/kg) and topical (1 mg/kg) administration of DCM extract inhibited oedema formation and reduced the neutrophil infiltration, which decreased the various parameters of the inflammatory response such as the hypertrophy of fibroblasts, papillomatosis, acanthosis, hyperkeratosis, and spongiosis [18]. The anti-inflammatory properties of these extracts have been explained with multi-faceted effects including, among others, a marked COX inhibitory activity, with a preferential inhibition of COX-2, inhibition of 5-LOX, iNOS, histamine and serotonine release, and leukocytic elastase, as previously demonstrated by Danz et al. [17,92].

In a model of delayed-type hypersensitivity (DHT) induced by dinitrofluorobenzene (DNFB) in mice, the lipophilic DCM extract, topically administered (1 mg/ear), inhibited the response both during the induction phase and the inflammatory phase. Instead, the oral administration had no effect [18].

Adjuvant-induced arthritis in rats is an experimental in vivo model mimicking the rheumatoid arthritis and it was utilized with the aim to evaluate the effect of a DCM extract on chronic inflammatory diseases [64]. In this model, the DMC extract (os, 150 mg/mL once/day, on days 17–23 by injection of the adjuvant) led to a significant reduction of paw oedema induced by injection of *Mycobacterium butyricum*. This effect did not result in a dose-dependency. A decreased joint damage, a reduction of tissular and articular inflammatory markers (oedema, cell infiltration, articular damage, pannus, cysts etc.), bone, and cartilage erosion was observed. Interestingly, the treatment over two weeks with a high dose of *I. tinctoria* extract did not determine macroscopic lesions of the rat gastric mucosa. The treatment did not cause a significant difference in tissue levels of COX-1 and COX-2, immunohistochemically determined. These results appear to disagree with those reported by Orberthür et al. [29] where a preferential inhibition of COX-2 and a reduced expression of iNOS was observed. In vitro studies demonstrated that the DCM extract is able to inhibit both tumour necrosis factor-α (TNF-α) and IL-β production in RAW 264,7 macrophages [64]. Thus, the anti-inflammatory and anti-allergic activity of *I. tinctoria* DCM extract highlighted in the arthritis and DTH models was related to the reduction of the production of these cytokines.

Brattström et al. [65] demonstrated the anti-inflammatory effects of SFE extract from the *I. tinctoria* leaves in a murine experimental model of allergic airway disease (asthma). The extract, administered intranasally (10–100 µg/mouse) to ovalbumin immunised BALB/c mice, determined a dose-dependent inhibition of the allergic reaction, which decreases the methacholine-induced airway hyperresponsiveness (AHR) and eosinophil recruitment, as determined both in the bronchoalveolar lavage (BAL) fluid and in the lung homogenates. The observed effect was related to the reduced production of the typical mediators of the Th2 immune response IL-4, IL-5, and RANTES (Regulated on Activation Normal T cell Expressed and Secreted) highlighted after treatment with *I. tinctoria* extract.

The anti-inflammatory activity of *I. tinctoria* was also evaluated in a clinical pilot study. The efficacy of the topical administration of different lipophilic extracts of *I. tinctoria* leaves was confirmed on healthy human volunteers using two experimental models, called sodium lauryl sulfate-induced irritant contact dermatitis and UVB-induced erythema. *I. tinctoria* extracts are effective when administered during the induction phase of dermatitis whereas they did not show any anti-inflammatory activity in the UVB-induced erythema model [63].

Various lipophilic constituents belonging to different chemical classes play an important role in the anti-inflammatory activity of *I. tinctoria* extracts such as the alkaloidis tryptanthrin, indirubin, indolinone, and fatty acids as linolenic acid.

Tryptanthrin possesses a unique pharmacological profile. It has been shown to be highly selective toward the COX-2 isoenzyme [17,92]. Nonetheless, other studies reported tryptanthrin as a non-selective COX inhibitor [93]. It inhibited 5-lipooxygenase (5-LOX) catalysed leukotriene synthesis in vitro and in vivo [18,92,94], and iNOS catalysed nitric oxide (NO) production [93]. In particular, tryptanthrin was found to be a potent natural inhibitor of cellular leukotriene biosynthesis in human whole blood and it is effective in vivo after oral administration in several experimental models such as in a murine model of inflammatory bowel disease (100 mg/kg p.o. for 3 days), in carrageenan-induced paw oedema in mice (50 mg/k p.o.), and in the rat pleurisy model (10 mg/kg p.o.) [18,94,95]. On the contrary, this compound, when topically administered, showed no significant anti-inflammatory effect in an animal model (TPA-induced ear oedema) [18] as well as in a clinical pilot study. In this study, carried out on healthy human volunteers, tryptanthrin, topically administered, did not manifest any protective effect both in the UVB-induced erythema model and in sodium lauryl sulfate-induced dermatitis [63]. Thus, the pure compound is not effective when administered by topical application. This observation was also confirmed by Oberthür et al. [62] through an experimental model of cutaneous micro-dialysis. It has been shown that the tryptanthrin contained in the extracts penetrates to a greater extent than that of pure solutions. This is because, in the first case, the alkaloid remains in molecular dispersion while, in the second case, it tends to crystallize on the surface of the skin.

One of the active compounds isolated from *I. tinctoria* leaves responsible for the anti-allergic properties is the alkaloid indolin-2-one. It has been demonstrated that indolinone inhibits compound 48/80-induced histamine release from rat peritoneal mast cells [96]. Moreover, indolinone was found to block IgE-mediated degranulation of sensitized mast cells at nM concentrations without directly interfering with signalling upstream of the histamine-containing granules [97]. 

Several studies demonstrated that indirubin is a potent inhibitor of cyclin-dependent kinase 5 (CDK5/P25) and glycogen synthase kinase-3-β (GSK-3-β) [98] as well as interferon-γ and interleukin (IL)-6 [99]. In the experimental model of LPS-induced pulmonary oedema in mice indirubin was shown to diminish oxidative stress and inflammation by reducing MDA production as well as IL-1β and TNF-α expression [100]. 

The analgesic potential of the lipophilic extract of *I. tinctoria* dried rosette leaves (DCM extract) was shown in vivo by an acetic acid–induced abdominal writhing test. The oral administration of the extract (200 mg/kg), before the intraperitoneal injection of acetic acid, reduced the number of writhing and stretching movements in mice while tryptanthrin (40 mg/kg) had no such effect [18].

### 5.2. Anti-Tumor Activity

*I. tinctoria* has been used in China to treat patients with solid tumours and leukaemia [9]. Phase I/II clinical trials highlighted very encouraging therapeutic effects of *I. tinctoria* in treating chronic myelocytic leukaemia [101].

Experimental studies with transplantable tumours demonstrated that *I. tinctoria* significantly prolonged the life span of Walker carcinosarcoma 256 bearing rats [101].

The anti-cancer properties of this species have been mainly attributed to the alkaloids’ indirubin and tryptanthrin, whose activity has been demonstrated by in vivo and in vitro experimental models. Numerous studies showed the potential efficacy of these compounds against a broad range of tumour types and the mechanisms of action underlying their activity have been elucidated.

The bisindole indirubin has been described about 40 years ago as being active in treating human chronic myelocytic leukaemia. Several trials published in China indicated that indirubin orally administered at the dose of 150–200 mg of per day led to remission in 60% of patients [9]. It was reported to induce complete remission in 26% and partial remission in 33% of 314 patients suffering from chronic myelocytic and chronic granulocytic leukaemia, which exhibited low toxicity and had limited adverse effects [66].

In animal leukaemia and lung carcinoma models, indirubin showed a good inhibitory effect and low toxicity [6]. It was found to inhibit DNA synthesis in rats bearing Walker-256 sarcoma [102]. A weak binding of indirubin to DNA has been demonstrated in vitro by Wu et al. [103] using the isotope labelling method, the spectrophotometric method, and thermal denaturation measurements, which shows that the binding between indirubin and DNA might be of a hydrogen bond rather than ionic. 

In an experimental study carried out by Zhang and colleagues [75], the treatment for 25 days with indirubin (10 mg/kg per day) has proven to be effective in inhibiting prostate tumour growth in a xenograft mouse model (BALB/c nude mice) by inhibiting tumour angiogenesis, as confirmed through a chick chorioallantois membrane (CAM) assay and a mouse corneal model (C57BL/6 mice). Western blot analysis highlighted that indirubin suppressed endothelial cell viability by blocking a vascular endothelial growth factor receptor 2- (VEGFR2) mediated Janus kinase (JAK)/STAT3 signalling pathway in endothelial cells. In particular, the phosphorylation and activation of JAK2 and STAT3 was suppressed, and the STAT3 downstream genes including Bcl-2, Bcl-xl, Cyclin D1, Cyclin A, and Survivin were down-regulated. The same authors also showed that indirubin effectively inhibited proliferation and induced apoptosis in human umbilical vein endothelial cells (HUVEC), while also inhibiting migration and capillary-structure formation. The core proteins involved in apoptosis including Caspase-3 and PARP were found to be activated.

The anti-tumour properties of indirubin and its derivatives appear to correlate with their antimitotic potential and inhibition of cyclin-dependent kinases (CDKs) and cell cycle regulatory molecules, which play an essential role in the regulation of cell proliferation, transcription, and apoptosis [70,104]. It was found that indirubin and its analogues inhibited the proliferation of the human large cell lung carcinoma LXFL529L and human mammary carcinoma MCF-7 cells. The more soluble analogue indirubin-3′-monoxime induced G2/M arrest in MCF-7 cells synchronized in the G2/M phase by a transient exposure to nocodazole and this effect was mediated by the inhibition of CDK1 and CDK1/cyclin B activity. This was suggested as a major mechanism by which indirubin derivatives exert their potent anti-tumour efficacy [70].

Indirubins are also efficient inhibitors of CDK2 and CDK5/p35. They act by competing with ATP for binding to the catalytic subunit of the kinase [102,105]. The crystal structure of the complex CDK2/indirubin derivatives showed that indirubin interacts with the kinase’s ATP-binding site through van der Waals interactions and three hydrogen bonds [102].

Another study published by Damiens et al. [106] reported that, after transient treatment with indirubin-3’-monoxime of human breast epithelial HBL-100 cells synchronized in G2/M by nocodazole, cells underwent an endoreplication. After the compound has been removed, the polyploid cells became aneuploid and, later, died from necrosis. This mechanism of endoreplication followed by cell death may contribute to the anti-tumour properties of indirubins.

The indole quinazolinone alkaloid tryptanthrin showed cancer chemo-preventive efficacy in intestinal tumour formation induced by azoxymethane in F344 rats [69]. It was found to be able to inhibit the growth of the murine myeloid leukaemia WEHI-3B JCS cells in the tumour-bearing BALB/c mice [73].

Recently, tryptanthrin has also been shown to be an effective suppressor of non-melanoma skin cancer (NMSC), as demonstrated by using dimethylbenz[a]anthracene/phorbol 12myristate 13acetate (DMBA/PMA) induced skin carcinogenesis model in *Swiss albino* mice [79]. It was found that the compound disrupted DMBA/PMA-induced expansion of hair follicle cells by suppressing the activation of β-catenin, which is a major driver of hair follicle cell proliferation. Additionally, tryptanthrin suppressed the activation of ERK1/2 and p38, in which both promote β-catenin activation and lowers the expression of c-Myc and cyclin-D1. These are the transcriptional targets of β-catenin, which are widely implicated in carcinogenesis.

Increasing evidence demonstrated that tryptanthrin is highly cytotoxic to a variety of a solid tumour and leukaemia cell lines and has a broad range of downstream targets that regulate tumour-associated cell processes including cell growth, cell cycle progression, and survival.

Tryptanthrin was found to inhibit the growth of human gastric cancer (HGC), lung cancer (HLC), and promyelocytic leukemia HL-60 cells with IC_50_ values of 1.5–4.2 μg/mL [67]. It showed significant cytotoxicity against non-small cell lung cancer NCI-H460, human glioblastoma SF-268, and human breast cancer MCF-7 cell lines with IC_50_ values of 8.5–22.6 μM [78]. Tryptanthrin also exhibited a multi-drug resistance reversing effect in doxorubicin-resistant breast cancer MCF-7 cells (MCF-7/adr), which is higher than that of the multi-drug resistance (MDR)-reversing agent Verapamil through down-regulation of *MDR1* gene expression, and partly by modulating the GSTpi-related pathway, which is a non-transporter pathway [71,72]. 

Liao and Leung [76] reported that tryptanthrin is able to inhibit the growth of the *N-myc* amplified human neuroblastoma LA-N-1 cells (IC_50_ = 15.8 ± 1.41 μM) as well as two other human neuroblastoma cell lines SH-SY5Y and SK-N-DZ, which induced cell cycle arrest at the G0/G1 phase. Western blot analysis showed a marked down-regulation of Cyclin D1, Cyclin D3, CDK4, and CDK6, which are known to be associated with the cell-cycle progression from the G1 to the S phase. LA-N-1 cells treated with tryptanthrin underwent marked morphologic differentiation, enhancement of acetylcholine esterase activity, and up-regulation of various differentiation markers including growth-associated protein 43 (GAP43), microtubule-associated protein tau (MAPT), calcitonin gene related peptide (CGRP), and somatostatin, with an effect superimposable to that of 5 μM 9-cis-RA used as a positive control. Moreover, tryptanthrin treatment led to a significant reduction of the N-myc proto-oncogene expression.

The anti-angiogenic activity has been proposed as an important mechanism underlying the anti-cancer properties of tryptanthrin against various solid tumours. A study published by Liao and colleagues [77] demonstrated that tryptanthrin inhibited angiogenesis both in vitro and in vivo. In particular, it inhibited the proliferation of human microvascular endothelial HMEC-1 cells and interrupted the migration and capillary-like structure formation. The anti-angiogenic effect was confirmed in vivo by a Matrigel plug assay. The underlying molecular mechanisms of action were explained by a reduced expression of pro-angiogenic factors such as Ang-1, PDGFB, and MMP2, suppression of the phosphorylation of VEGFR2, and blockade of the VEGFR2-mediated ERK1/2 signalling pathway. Molecular docking studies indicated that tryptanthrin could bind to the ATP-binding site of VEGFR2.

Concerning the antileukemic activity, at low concentrations (0.5 μg/mL), tryptanthrin was found to be able to enhance the expression of cell differentiation markers in human monocytic U-937 and promyelocytic HL-60 leukaemia cells, which is indicative of their differentiation into monocytes and macrophages. Higher concentrations inhibited cell proliferation and induced cell death by apoptosis in the cultures by damaging the mitochondria that induces the apoptotic cascade through a caspase-3/Fas antigen pathway [68]. 

Chan et al. [73] reported the potent anti-proliferative efficacy of tryptanthrin against murine myeloid leukaemia WEHI-3B JCS cells, with an IC_50_ value of 1.5 μM at 48 h of treatment. Flow cytometric analysis showed the cell cycle arrest of the cells at the G0/G1 phase in a dose-dependent manner. The expression of the cell cycle-related genes including cyclin D2, D3, Cdk 2, 4, 6 was found to be significantly down-regulated at 24 h as measured by real time PCR, which led us to propose the prevention of the cell cycle progression from the G1-phase into the S-phase. This causes cell cycle arrest as one of the mechanisms for leukemic cell growth inhibition. Furthermore, it has been suggested that tryptanthrin exerts its anti-tumor effect on WEHI-3B JCS cells by triggering cell differentiation, as demonstrated by morphological and functional studies.

Miao et al. [74] had proved that tryptanthrin significantly inhibited human chronic myeloid leukaemia K562 cell proliferation with an IC_50_ value of 8.8 μg/mL after 48 h of treatment. Cell cycle distribution analysis showed that it inhibited proliferation by blocking the cell cycle progression at the G0/G1 phase and, subsequently, progressing into apoptosis. The underlying mechanism has been attributed to the damage of mitochondrial membrane and the cyt-c-caspase-3 dependent mechanisms.

Besides the lipophilic compounds indirubin and tryptanthrin, the polar phenolic constituents have also been shown to contribute to the anti-cancer properties of *I. tinctoria*. Recently, the anti-proliferative properties of polar extracts obtained from *I. tinctoria* rosette leaves together with those from the cauline leaves and the flowers have been evaluated [47]. To achieve the separation of the polar compounds from the lipophilic ones, frozen and lyophilized plant material was sequentially extracted with dichloromethane and 70% methanol. *I. tinctoria* polar extracts showed anti-proliferative effects against the human anaplastic thyroid cancer ATC cell lines CAL-62, 8505C and C-643. Particularly, after 48 h of treatment, the basal leaf extract markedly inhibited the growth of CAL-62 cells, which caused nearly 85% reduction of viability at the highest tested dose (1 mg/mL). The significant anti-proliferative effects against ATC cell lines have been related to the flavonoids and the phenolic acids contained in the extracts, characterized by HPLC-PDA/ESI-MS. Another study published by the same authors confirmed that phenolics were mainly responsible for the observed activity. The anti-proliferative properties of the phenolic-rich fraction obtained from *I. tinctoria* cauline leaf polar extract has been demonstrated, which caused 80% and 65% growth inhibition in CAL-62 and 8505C cells, respectively, after 48 h of exposure at the maximum tested dose (0.1 mg/mL). The activity of the phenolic-rich fraction against these cell lines was similar to that of Sunitinib used as a positive control, when tested at the dose of 10 μM for 72 h [46].

### 5.3. Antimicrobial and Antiviral Activities

A few research studies investigated *I. tinctoria* as a potential source of valuable compounds with anti-microbial efficacy against bacterial and fungal species. More thorough studies have been conducted on components with antiviral activity, which have led to the identification of possible mechanisms of action (Table 2). 

The results of a study carried out by Dornberger and Lich [81] on the anti-microbial activity of a hydroalcoholic (water/ethanol) extract of the fresh whole plant evaluated by an agar diffusion test indicated that, among the 23 tested strains (Gram-positive, Gram-negative, yeast, and fungi), only the Gram-positive tetracycline-resistant *Bacillus mycoides* SG 756 TF, *B. subtilis* ATCC 6633, and *Micrococcus luteus* SG 125 A, and the yeast *Saccharomyces cerevisiae* JH 3 showed weak sensitivity to the extract. By contrast, Ullah and colleagues [85] recently reported the good anti-microbial properties of different extracts from *I. tinctoria*. In particular, the study concerned the evaluation of anti-microbial activity of fractions obtained from different parts of the plant (branches, flowers, leaves, and roots) by extraction with 14 different solvents, which was performed using a micro-titer plate method against seven bacterial and four fungal strains. The obtained results showed that the extracts displayed antibacterial activity, more elevated against Gram-positive strains (*B. subtilis, M. luteus and Staphylococcus aureus*). In many cases, it was higher than the antibiotic cefotaxime used as a positive control as well as antifungal activity (except for branches). In general, the leaves were found to be the best plant part and ethyl acetate, n-hexane, chloroform, and acetone as the most efficient solvents for the extraction of antimicrobial compounds.

The possible utilization of *I. tinctoria* in combination therapy with commonly used antibiotics for treating infections caused by meticillin-resistant *S. aureus* (MRSA), which is a widespread cause of both community-acquired and hospital-acquired infections associated with significant mortality and morbidity, has been suggested. The dried leaf hydroalcoholic (75% ethanol) extract of *I. tinctoria* was found to be able to potentiate the effects of four antibiotics (penicillin G, gentamicin, ciprofloxacin, and ceftriaxone) against both MRSA (isolated from hospital patients) and standard (ATCC25923) *S. aureus* strains [82]. 

Among the phytochemicals contained in *I. tinctoria*, the alkaloid tryptanthrin has been proven to have an antimicrobial effect. Nonetheless, there are discrepancies between the results of various research studies. One of the earliest published works is that of Honda and colleagues, who evaluated the activity of tryptanthrin against a wide variety of microorganisms including bacteria, yeasts, dermatophytes by the agar dilution streak method, and phytopathogens by the paper disc method. The results of these investigations demonstrated that it displays strong activity against *Bacillus subtilis* (MIC: 25 μg/mL) and *B. polymyxa* (MIC: 50 μg/mL), and it is a highly specific antimicrobial agent against dermatophytes, which are causative of athlete’s foot, namely *Trichophyton mentagrophytes*, *T. rubrum* IFO 5808, *T. tonsurans* var. *sulfureum* IFO 5945, *Microsporum canis* IFO 9182, *M. gypseum* IFO 8307, and *Epidermophyton floccosum* IFO 9045 (MICs: 3.1–6.3 μg/mL) [43,80]. By contrast, the results of the agar diffusion assay carried out by Chiang et al. [83] revealed that tryptanthrin showed no effect on tested fungi including the dermatophytic pathogens *E. floccosum* ATCC 18397, *M. gypseum* ATCC 14683, and *T. rubrum* ATCC 10218. The same authors reported that tryptanthrin significantly (≥12.5 μg/disc) inhibited the growth of *S. epidermis* ATCC 12228 and *S. aureus* ATCC 6538, and only weakly suppressed MRSA ATCC 43300 even at 100 µg/disc. This last result is in disagreement with that of a more recent study, which highlighted a promising anti-bacterial activity of tryptanthrin against MRSA, as evaluated by the agar diffusion method (MIC 62.5 μg/mL) [84]. 

The traditional Chinese medicine *Bǎn Lán Gēn* (Isatidis radix) is widely employed in the prevention and treatment of a wide range of viral infections including seasonal flu, the deadly Severe Acute Respiratory Syndrome (SARS), viral pneumonia, mumps, pharyngitis, and hepatitis [87,107]. Different types of compounds effective against influenza viruses have been isolated from I. radix. Indirubin has been shown to have powerful activity vs. the influenza virus A/NWS/33- and B/Lee/40-infected bronchial epithelial cells H292 by reducing both the expression and production of the chemokine RANTES [86].

The polysaccharide compounds contained in I. radix also have anti-influenza virus activity, as demonstrated by in vitro investigations. It has been reported that a polysaccharide isolated from I. radix inhibited the attachment of the influenza virus to the red blood cells, which promoted the generation of anti-influenza viral IgG antibodies [88]. In a study carried out by Li et al. [87], I. radix polysaccharides showed potent anti-influenza A virus activity against human seasonal influenza viruses (H1N1 and H3N2) and avian influenza viruses (H6N2 and H9N2). They were found to effectively inhibit the expression of pro-inflammatory cytokines (IL-6) and chemokines (IP-10, MIG, and CCL-5) induced by the human influenza virus PR8/H1N1 and the avian influenza virus H9N2. Furthermore, the polysaccharides reduced the expression of the Toll-like receptor (TLR)-3 induced by the PR8/H1N1 virus. These results led to hypothesize that the underlying mechanism of action of the polysaccharides is to impair the upregulated expression of pro-inflammatory cytokines/chemokines induced by the influenza virus by inhibiting the expression of host TLR-3. 

The in vitro antiviral efficacy of the I. radix polysaccharide against type II herpes simplex virus (HSV-2) has been recently demonstrated [88]. Using the 3-(4,5-dimethylthiazole-2-yl)-2,5-diphenyltetrazolium bromide (MTT) assay, this effect was shown to occur mainly by inhibiting the viral duplication and adsorption.

### 5.4. Antioxidant Activity

In the last decade, some research has focused on the study of *I. tinctoria* as a source of natural antioxidants. The first published articles concerning the antioxidant properties of *I. tinctoria* extracts were those of Fialova et al. and of Yang et al. [89,90]. In the first work carried out on a hydroalcoholic extract of the leaves, good radical scavenging activity in the 1,1-diphenyl-2-picrylhydrazyl (DPPH) test was found (SC_50_ = 103.9 µg/mL) whereas the second one reported very weak activity in the same test as well as in the Trolox Equivalent Antioxidant Capacity (TEAC) and in reducing power assays for an ethanol extract obtained from dried Chinese *I. tinctoria*.

The results of subsequent investigations on *I. tinctoria* leaves indicated that phytochemicals belonging to different chemical classes act as antioxidants, such as alkaloids and phenolic compounds. 

In a study carried out by Zhao and collegues [91], the antioxidant properties of the indole alkaloids indigo and indirubin were evaluated by means of different in vitro assays. Indigo and indirubin were found to be more effective for superoxide anion scavenging (EC_50_ = 0.61 mg/mL and 0.74 mg/mL, respectively). This effect has been attributed to the presence of some electrophilic groups, such as keto or aldehyde, which facilitate the release of hydrogen from the O–H bond and stabilize the superoxide anion. Both compounds exhibited scavenging activities on a DPPH-free radical, whereas they did not show hydroxyl radical (OH•) scavenging activity and reducing power.

It is well known that polyphenols represent the main class of natural antioxidants, which act in different ways such as radical scavengers because phenolic groups are excellent nucleophiles and are also able to inhibit lipid peroxidation, as breakers of the oxidation reaction by binding with free radicals generated through lipid peroxidation, and as chelators of metal ions that induce oxidation [108]. Aimed at establishing the involvement of phenolic compounds in the antioxidant properties of *I. tinctoria*, recently, the polar extract obtained from the rosette leaves and also those from the cauline leaves and the flowers have been investigated by in vitro assays based on different mechanisms [47]. Lyophilized plant material was sequentially extracted with dichloromethane and 70% methanol in order to separate the polar compounds from the lipophilic ones. The polar extracts were found to possess good radical scavenging (DPPH test) and ferrous ions’ chelating activities and moderate reducing power, and those from cauline leaves and flowers displayed better antioxidant efficacy than the rosette leaf extract. The calculated coefficient of determination (R^2^) indicated that flavonoids were the phenolic compounds mainly involved in the observed antioxidant properties. Another study carried out by the same authors on the phenolic-rich fraction obtained from *I. tinctoria* cauline leaf polar extract confirmed that phenolics were mainly responsible for the good radical scavenging properties [46]. In particular, the activity has been mainly related to the flavonols detected in the fraction, which have all the essential structural elements for potent radical scavenging activity. Compared to flavone compounds, the flavonols contain more hydroxyl groups (one to six) among which the OH group at the C-3 position has a very high ability to scavenge the DPPH radical, such as quercetin and rutin, which are well-known potent antioxidants. Interestingly, these works have highlighted the antioxidant activity not only of the rosette leaves, which are commonly utilized for their medicinal properties, but also of the cauline leaves and the flowers, which indicates a broader range of usage possibilities for this relevant species. 

Even the roots of *I. tinctoria* are a source of antioxidant compounds. Indeed, the polysaccharides contained in the roots were found to possess antioxidant properties in vitro. In particular, in a study conducted by Han et al. [34], the polysaccharides extracted from the dried roots exhibited an appreciable radical scavenging activity in the 2,2-azino-bis-3-ethylbenzothiazoline-6-sulfonic acid (ABTS) assay, reaching, at the maximum tested concentration (0.3 mg/mL), a percentage of inhibition (64.3%) close to that of vitamin C used as a reference compound. 

## 6. Plant Biotechnological Studies on *I. tinctoria*

It has been reported that the phytochemical profile of *I. tinctoria* field-cultivated is highly affected by various factors including cultivar, geographical regions, climatic fluctuations, and soil conditions [109].

The techniques concerning plant in vitro cultures and the possibilities of increasing the accumulation of secondary metabolites established by plant biotechnology are a known, alternative source of many valuable biologically active secondary metabolites used in medicine as well as in cosmetology and the food industry [110,111]. The many strategies used in plant biotechnology aimed at increasing the in vitro culture biomass and/or bioactive metabolites’ production. The elaboration of micropropagation protocols creates the possibilities of increasing and facilitating the specific, often very demanding, plant multiplication [112]. For increasing the secondary metabolites production in in vitro cultured biomass, different, specific strategies have been introduced [113]. The most important ones are: the selection of high productivity cell lines, elaboration of the optimal culture conditions (e.g., selection of basic formulation of the culture medium, type of plant growth regulators, and their concentrations in the media, types of cultures, and optimal lighting and temperature conditions), establishment of cultures with a high degree of organogenesis (shoot, root cultures), application of biotic or abiotic elicitors, and genetic transformation (e.g., hairy root cultures). Increasing the biomass production and the scale-up of cultures’ production in the special plant bioreactors are also important aspects of biotechnology studies [111,114,115].

The growing interest in the beneficial values of *I. tinctoria* has been concentrated mainly on the roots of this plant as the best source of demanding metabolites. As apparent from the analysis of the latest scientific literature, the biotechnological studies on *I. tinctoria* are limited mainly to hairy root cultures. Interestingly, within the last four years, the teams of two researchers: Gai and Jiao from Northeast Forestry University, Harbin in China, have published a series of research publications on *I. tinctoria* hairy root cultures. The cultures were successfully initiated from in vitro petiole explants co-cultured with *Agrobacterium rhizogenes* with the addition of acetosyringone and arginine. In the obtained hairy root cultures, the high production of alkaloids [116] and flavonoids [117] was confirmed. Within the alkaloids epigoitrin, isatin, indole-3-carboxaldehyde, tryptanthrin, indigo, and indirubin were qualitatively and quantitatively determined by LC–MS/MS. The total alkaloid content (521.77 μg/g DW (dry weight)) was 1.12 times higher than in two-year-old roots of field grown plants (464.69 μg/g DW) [116]. Under the study on flavonoid composition, eight bioactive constituents were estimated by the LC-MS/MS method: rutoside, neohesperidin, buddleoside, liquiritigenin, quercetin, isorhamnetin, kaempferol, and isoliquiritigenin [117]. Under optimal conditions, the total flavonoid content (438.10 μg/g DW) was found to be 1.28 times higher than in two-year-old field grown roots (341.73 μg/g DW) analyzed for comparison. Additionally, in vitro antioxidant assays demonstrated that hairy root extracts exhibited higher activities than those from roots of field grown plants [117]. 

Such promising results prompted the Chinese team to perform the elicitation experiments on these cultures in order to boost the bioactive metabolites’ production. The elicitation experiment with chitosan was proven to increase the flavonoid yield in *I. tinctoria* hairy root biomass [118]. In comparison with the control (2.31 mg/g DW), a 7.08-fold enhancement of total flavonoids (16.35 mg/g DW) was achieved in 24-day-old hairy root cultures elicited by 150 mg/L chitosan for 36 h [118]. Under another study, the *I. tinctoria* hairy root cultures were exposed to ultraviolet radiation (UV-A, UV-B, and UV-C) in an attempt to promote the production flavonoids. Results showed that the maximum flavonoid accumulation (7259.12 μg/g DW) in hairy root cultures treated by 108 kJ/m^2^ dose of UV-B radiation increased 16.51-fold as compared to the control (439.68 μg/g DW) [119]. Moreover, the same teams proved the increase of flavonoid production after *I. tinctoria* hairy root cultures co-cultivation with two immobilized live GRAS (Generally Recognized as Safe) fungi: *Aspergillus niger* and *Aspergillus oryzae*. *A. niger* strains were exhibited as the better biotic elicitor in the plant-fungus co-cultivation system. The highest flavonoid production (3018.31 μg/g DW) was achieved after treatment with spores at the concentration of ca.104 spores/mL, temperature of 30 °C, pH 7.0, and time of 72 h. The total amount of flavonoids increased 6.83 times in comparison to control hairy roots (441.91 μg/g DW) [120]. A recent work on elicitation of *I. tinctoria* hairy root cultures proved enhanced production of flavonoids as well as alkaloids after abiotic elicitation protocol applied with salicylic acid and methyl jasmonate [121]. As determined by central composite design based on a mathematical model, the maximum accumulation of alkaloids was found in biomass elicited by 142.61 µM salicylic acid for 28.18 h and flavonoids in biomass elicited by 179.54 µM methyl jasmonate for 41.87 h. These amounts increased 5.89-times and 11.21-times, respectively, in comparison with the control. 

The above described biotechnological studies on *I. tinctoria* hairy roots are a good example of the importance of optimization culture conditions, aimed at high secondary metabolites’ production. Authors claim that their research studies are a promising and effective approach for the enhanced production of flavonoids and alkaloids in *I. tinctoria* in vitro cultures, which allows for the improved applicability of these valuable compounds in pharmaceutical fields, and that their studies pave the way toward the successful commercialization of this culture system in the future. 

In the available scientific literature, there is only one publication on the development of the micropropagation protocol for *I. tinctoria*, based on the adventitious shoot regeneration method. Under the work of Saglam and Ciftci [122], the effective shoot regeneration system was developed using ‘isubgol’ as a cheap alternative gelling substance. The leaf and hypocotyl explants were cultured on Murashige and Skoog (MS) [123] medium solidified with 6.5 g/L agar or 15 g/L isubgol containing different concentrations of BA (6-benzyladenine) and NAA (1-naphthaleneacetic acid). Maximum regeneration of 12.65 and 17.80 shoots per leaf explant was observed on MS medium solidified with agar containing 1 mg/L BA with 0.25 mg/L NAA and on MS medium solidified with isubgol containing 0.50 mg/L BA, respectively. Maximum regeneration of 19.87 and 20.55 shoots per hypocotyl explant on agar and isubgol gelled media was recorded on MS medium containing 0.50 mg/L BA with 0.25 mg/L NAA, and 1 mg/L BA, respectively. The gelling agent Isubgol gave better shoot regeneration as compared to agar. 

In the existing literature, only one study on shoot cultures of *I. tinctoria* is available. In particular, Reference [124] obtained the establishment of *I. tinctoria* shoot cultures starting from the nodal segments of young plants cultured on MS and Gamborg (B5) [125] media supplemented with BA or KIN (kinetin) with or without NAA. The best multiplication rates were obtained on B5 medium supplemented with 1 mg/L BA (3.4 shoots per explant). The addition of NAA to the tested media did not increase multiplication. 

**Figure 8:**
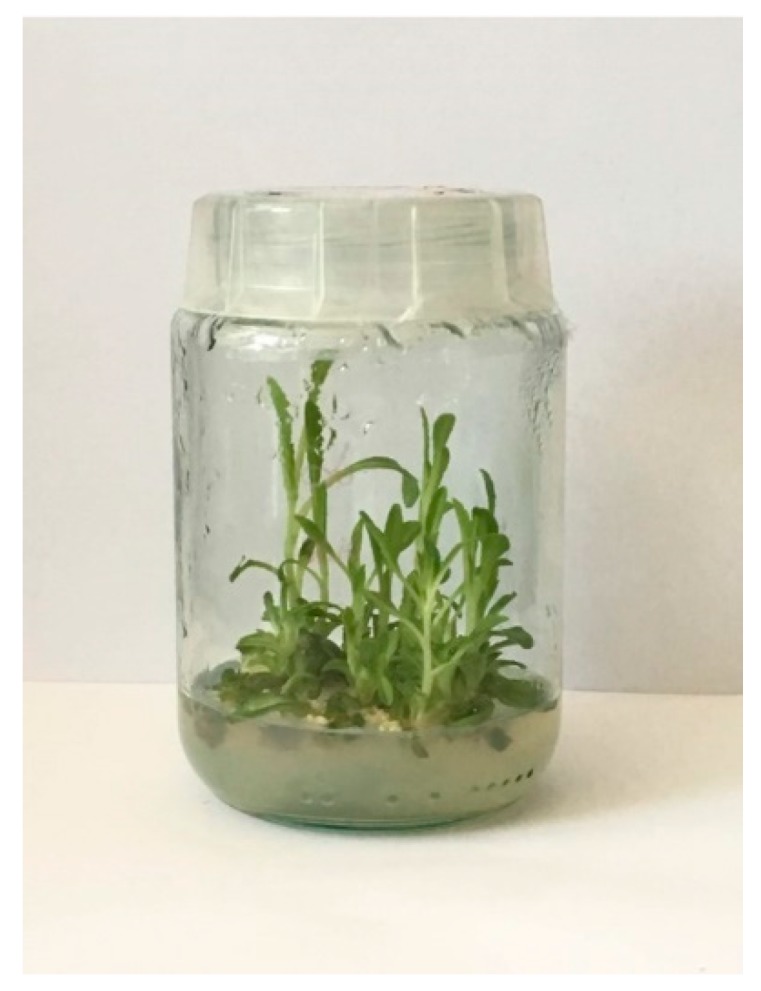
The microshoot in vitro cultures of *I. tinctoria* L. (agar Murashige and Skoog medium with 1 mg/L BA and 1 mg/L NAA) after two weeks of growth.

Currently, under the cooperation of our teams, from the Department of Chemical, Biological, Pharmaceutical and Environmental Sciences, University of Messina (Messina, Italy), and the Department of Pharmaceutical Botany, Jagiellonian University, Collegium Medicum (Kraków, Poland), in vitro shoot cultures of *I. tinctoria* have been successfully initiated, which aimed to investigate whether they could represent a potential source of bioactive compounds (Figure 8) [unpublished]. The cultures were successfully initiated from the seeds, and the preliminary media composition optimization was performed. The MS media variants with BA and KIN as cytokinins and NAA and IBA (indole-3-butyric acid) as auxins in different concentrations (range from 0 to 2 mg/L) were tested. The best conditions for agar shoot cultures were proven to be MS medium containing 1 mg/L BA and 1 mg/L NAA on which the biomass increment was equal to 3.58-fold (fresh weight).

Intensive biotechnological studies on *I. tinctoria* shoot culture are in progress and are aimed at obtaining high production of secondary metabolites in the biomass (as well as in the growth media) such as phenolic acids or glucosinolates, which were never studied before.

## 7. Conclusions

*I. tinctoria* has an ancient and well-documented history as an indigo dye and medicinal plant. It has been long utilized as an ornamental crop and an animal feeding plant, and, currently, its importance increased in cosmetic industry. Due to the relevance of this species, a large number of in vitro and in vivo investigations carried out on *I. tinctoria* mostly in the last 20 years have provided reasonable support for its various traditional uses, which shows that the diverse extracts or compounds isolated from different parts of this species have a broad spectrum of biological activities. As far as we know, there is only one previous review article by Hamburger (2002), which reported studies published from 2000 to 2002 focusing only on the anti-inflammatory activity of *I. tinctoria* extract and its active component tryptanthrin.

This article review provides an up-to-date and comprehensive overview mainly on the phytochemistry and the biological properties demonstrated for this valuable species in order to support its therapeutic potential and to offer input for future research prospects.

The article also focuses on all attempts in plant biotechnology studies for the enhanced production of bioactive compounds from *I. tinctoria* hairy root and shoot cultures as an alternative to plant raw materials. Plant in vitro culture technology has emerged as an attractive alternative for the field cultivation of *I. tinctoria*. Due to the high therapeutic value of *I. tinctoria*, the biotechnological approach represents a promising way to develop safe and more effective extracts than traditional ones. The large-scale production of biotechnological extracts, enriched in desired metabolites and obtained by different in vitro systems, should become the goal of further scientific studies.

## Figures and Tables

**Table 1:** Phytochemical composition of *I. tinctoria* L.

Chemical Class	Compound	Site of Collection	Plant Part/Extract	Ref.
**Alkaloids**	Tryptanthrin	Japan	Dried rosetta leaves Chloroform	[43]
	Isatin	France	Fresh Leaves Acetone/acetic acid 1% v/v	[44]
	Isatan A – B – C			
	Isoindigo			
	Indoxyl			
	Indicant			
	*cis/trans* Indirubin			
	*cis/trans* Indigo			
	(*E*)-3-(3’,5’-Dimethoxy-4’-hydroxy-benzylidene)-2-Indolinone	Germany	Dried rosette leaves Dichloromethane	[33]
	5-Hydroxyoxindole			
	3-(2’-Carboxyphenyl)quinazolin-4-one			
	Bisindigotin			
	Deoxyvasicinone			
	N-formyl anthranilic acid	Germany	Dried rosette leaves Methanol	[33]
	Acetylindican-carboxyl acid	France	Frozen and lyophilized rosette leaves Methanol	[16]
	6-Hydroxyindolone-3-carboxylic acid 6-O-glucoside	France	Frozen and lyophilized/dried rosette leaves Methanol and Dichloromethane	[45]
	6-Hydroxyindolone-3-carboxylic acid glucose ester			
	Acetylindican			
	Malonylindican			
	Dioxindole glucoside			
	Dyhydroascorbigen			
**Flavonoids and their conjugates**	Vicenin-2	Germany	Dried rosette leaves, Methanol	[33]
	Stellarin-2			
	Isoorientin			
	Isovitexin			
	Isoscoparin			
	Isoorientin-3”-O-glucoside			
	Isovitexin-3”-O-glucoside			
	Isoscoparin-3”-O-glucoside			
	Isoscoparine	Germany	Dried rosette leaves Dichloromethane	[33]
	Luteolin-6-C-glucoside-7-O-glucoside	France	Frozen and lyophilized rosette leaves Methanol	[16]
	Vicenin-2			
	Stellarin-2			
	Isovitexin			
	Isovitexin-3”-O-glucoside			
	Isovitexin-3”-O-glucoside-7-O-glucoside			
	Isoorientin			
	Isoorientin-3”-O-glucoside			
	Iscoscoparin			
	Isoscoparin-3”-O-glucoside-7-O-glucoside			
	Iscoscoparin-3”-O-glucoside			
	4’-O-Feruloyl iscoscoparin-3”-O-glucoside-7-O-glucoside			
	2”-O-Feruloyl isoscoparin-3”-O-glucoside-7-O-glucoside			
	Isoscoparin-3”-O-glucoside-7-O-feruloylglucoside			
	Isoscoparin-3”-O-*p-*coumaroylglucoside			
	Isoscoparin-3”-O-sinapoylglucoside			
	Isoscoparin-3”-O-feruloylglucoside			
	Chrysoeriol-7-O-glucoside			
	Luteolin glucuronide	Italy	Lyophilized cauline leaves 70% Methanol	[46]
	Rutin			
	Vicenin-2			
	Bluddleoside			
	Stellarin-2			
	Flavone-di-glucoside			
	Apigenin-di-glucoside			
	Isovitexin			
	Quercetin			
	Isoscoparin			
	Isoscoparin-di-glucoside			
	Kaempferol			
	Apigenin-glucoside			
	Luteolin- glucuronide	Italy	Lyophilized rosette leaves 70% Methanol	[47]
	Vicenin-2			
	Stellarin-2			
	Flavone-di-glucoside			
	Apigenin-glucoside			
	Apigenin-di-glucoside			
	Isovitexin			
	Quercetin			
	Isoscoparin			
	Luteolin-glucuronide	Italy	Lyophilized cauline leaves 70% Methanol	[47]
	Flavone-di-glucoside			
	Vicenin-2			
	Buddleoside			
	Stellarin-2			
	Apigenin-glucoside			
	Apigenin-di-glucoside			
	Isovitexin			
	Quercetin			
	Isoscoparin			
	Luteolin-glucuronide	Italy	Lyophilized flowers 70% Methanol	[47]
	Vicenin-2			
	Stellarin-2			
	Apigenin-di-glucoside			
	Isovitexin			
	Quercetin			
**Phenolic acids and their conjugates**	*p*-Hydroxybenzoic acid		Dried leaves, Methanol	[48]
	*o*-Methoxybenzoic acid			
	*p*-Methoxybenzoic acid			
	Dihydrocaffeic acid			
	4-Hydroxy-3-methoxyphenylpropanoic acid			
	Sinapic acid	Germany	Dried rosette leaves, Dichloromethane	[33]
	Ferulic acid			
	Neochlorogenic acid	Italy	Lyophilized cauline leaves 70% Methanol	[46]
	Chlorogenic acid			
	Caffeic acid			
	Coumarylquinic acid			
	Sinapic acid			
	Ferulic acid			
	*p*-Coumaric acid			
	Protocatechuic acid hexoside	France	Frozen and lyophilized rosette leaves, Methanol	[16]
	Protocatechoyl glucose			
	*p-*Coumaroyl glucaric acid			
	*p-*Coumaric acid hexoside			
	*p*-Coumaroyl dihexoside			
	*p*-Coumaroyl hexoside			
	*p-*Coumaroyl sinapoyl glucaric acid			
	di-*p*-Coumaroyl glucaric acid			
	Feruloyl dihexoside			
	Feruloyl glucaric acid			
	Diferuloyl glucaric acid			
	Feruloyl glicerate			
	Feruloyl sinapoyl glucaric acid			
	Feruloyl *p-*coumaroyl glucaric acid			
	Sinapoyl hexoside			
	Sinapoyl gentiobioside			
	Sinapoyl glucaric acid			
	Disinapoyl glucaric acid			
	Sinapoyl malate			
	Disinapoyl methoxyglucaric acid			
	Disinapoyl hexoside			
	Guaiacyl(8-O-4)feruloyl sinapoyl glucarid acid			
	Neochlorogenic acid	Italy	Lyophilized rosette leaves 70% Methanol	[47]
	Caffeic acid			
	Sinapic acid			
	Ferulic acid			
	Neochlorogenic acid	Italy	Lyophilized cauline leaves 70% Methanol	[47]
	Caffeic acid			
	Sinapic acid			
	Ferulic acid			
	Caffeic acid	Italy	Lyophilized flowers 70% Methanol	[47]
	Ferulic acid			
**Monolignols and oligolignols**	Syringe	France	Frozen and lyophilized rosette leaves Methanol	[16]
	Coniferin			
	Pinoresinol dihexoside			
	Syringaresinol hexoside			
	Isodihydrodehydrodiconiferyl alcohol hexoside			
	Isodihydrodehydrodiconiferyl alcohol dihexoside			
	5-Hydroxy-coniferyl alcohols hexoside			
	Syringyl(8-5)guaiacyl hexoside			
	Guaiacyl(8-5)guaiacyl hexoside			
	Guaiacyl(*erythro*8-O-4)guaiacyl hexoside			
	Guaiacyl(*threo*8-O-4)guaiacyl hexoside			
	Guaiacyl(*threo*8-O-4)dihydroguaiacyl hexoside			
	Guaiacyl(*threo* 8-O-4)syringyl(8-5)guaiacyl hexoside			
**Alifatic Glucosinolate**	Epiprogoitrin	Germany	Frozen and lyophilized rosette leaves 70% Methanol	[49]
	Progoitrin			
	Gluconapin			
**Indolic Glucosinolates**	Glucobrassicin	Germany	Frozen and lyophilized rosette leaves 70% Methanol	[49]
	Neoglucobrassicin			
	Sulfoglucobrassicn			
	4-Hydroxyglucobrassicin			
	Glucotropaeolin			
	Glucoisatisin/epiglucoisatisin	Germany	Seed Aqueous	[50]
	Gluconapoleiferin	France	Frozen and lyophilized rosette leaves Methanol	[16]
	Glucoibericin	Italy	Lyophilized rosette and cauline leaves, flowers 70% Methanol	[47]
	4-Methoxyglucobrassicin	Italy	Flower 70% Methanol	[47]
**Carotenoids**	(all-*E*)-β-Carotene	Germany	Dried rosette leaves Dichloromethane	[33]
	(13Z)-or (13’*Z*)- Lutein mixture	Germany	Dried rosette leaves Hexane/Acetone (1:1)	[33]
	(all-E)-Lutein			
	(9Z)-Lutein			
	(9’Z)-Lutein			
	(15Z)-β-Carotene			
	(9Z)-β-Carotene			
	(Z)-Neochrome			
	(15Z)-Violaxantin			
	(all-E)-Neochrome			
	(di-Z)-Violaxantin			
**Porphyrins**	10-Hydroxy phaeophorbide	Germany	Dried rosette leaves Dichloromethane	[33]
	Phaephorbide a			
	Phaephorbide a’			
	Pyrophaeophorbide a			
**Isothiocyanates and thiocyanates**	2-Hydroxy-3-butenyl isothiocyanate	Italy	Fresh leaves HS-SPME	[19]
	3-Butenyl isothiocyanate			
	Allyl isothiocyanate			
	Pentyl isothiocyanate			
	3-Methylthiopropyl isothiocyanate			
	Hexyl isothiocyanate			
	Benzyl isothiocyanate			
	Methyl thiocyanate			
	3-Butenyl isothiocyanate	Italy	Dried roots HS-SPME	[51]
	Ciclopentyl isothiocyanate			
	Methyl thiocyanate			
**Aldehydes**	3-Methylbutanal	Italy	Fresh leaves HS-SPME	[19]
	But-2-enal			
	Hexenal			
	*trans*-Pent-2-enal			
	*trans*-Hex-2-enal			
	Nonanal			
	*trans, trans*-Hexa-2,4-dienal			
	*trans*-Oct-2-enal			
	*trans, trans*-Hepta-2,4-dienal			
	Benzenecarbaldehyde			
	*cis, trans*-Nona-2,6-dienal			
	4-Ethylbenzenecarbaldehyde			
	Tetradecanal			
	Furfural	Italy	Dried roots HS-SPME	[51]
	Benzaldehyde			
**Sulfurated compounds**	2-Ethylthiophene	Italy	Fresh leaves HS-SPME	[19]
	Carbonyl sulphide			
	Carbon disulphide			
	Cyclopenthanethiol			
	Thiophene			
**Alcohols**	Tetradecan-1-ol	Italy	Fresh leaves HS-SPME	[19]
	2-Cyclopentylethanol			
	Butan-1-ol			
	*cis*-Pent-2-en-1-ol			
	*trans*-Hex-3-en-1-ol			
	2-Butyloctan-1-ol			
	Pentadecan-1-ol			
	Heptadecan-1-ol			
	2-Methylexadecan-1-ol			
	Nonadecan-1-ol			
	Hexanol	Italy	Dried roots HS-SPME	[51]
	1-Octen-3-ol			
	Heptanol			
	Furfuryl alcohol			
	2-Penylethyl alcohol			
	Phenol			
**Terpenes and Sesquiterpenes**	Limonene	Italy	Fresh leaves HS-SPME	[19]
	Sabinene			
	δ-3-Carene			
	Eucalyptol			
	γ-Terpinene			
	*p-*Cymene			
	Terpinolene			
	Myrtenal			
	*p-*Cymenene			
	β-Cyclocitral			
	Valencene			
	δ-Cadinene			
	Geranyl acetone			
	6-Methyl-5-hepten-2-one	Italy	Dried roots HS-SPME	[51]
	Camphor			
	Geranyl acetone			
	Guaiacol			
**Acids**	Acetic acid	Italy	Fresh leaves HS-SPME	[19]
	Octanoid acid			
	Butyric	Italy	Dried roots HS-SPME	[51]
	Octanoic acid			
**Esters**	Methyl-2-hydroxybenzoate	Italy	Fresh leaves HS-SPME	[19]
	Butyl tetradecanoate			
	Methyl butyrate	Italy	Dried roots HS-SPME	[51]
**Ethers**	1-Methoxy-4-prop-2-enylbenzene	Italy	Fresh leaves HS-SPME	[19]
	Dyphenil ether			
**Furans**	2-Ethylfuran	Italy	Fresh leaves Leaf HS-SPME	[19]
**Hydrocarbons**	*trans*-1,5-Heptadiene	Italy	Fresh leaves HS-SPME	[19]
	Heptadecene			
	Nonadecene			
	Eicosene			
	Heneicosene			
	Tetracosene			
	Decane	Italy	Fresh leaves HS-SPME	[51]
	Tridecane			
	Pentadecane			
	Heptadecane			
**Ketones**	1-Penten-3-one	Italy	Fresh leaves HS-SPME	[19]
	Octan-2,5-dione			
	*trans*-β-Ionone			
	2-Heptanone	Italy	Dried roots HS-SPME	[51]
	2-Nonanone			
	(E,E)-3,5-Octadien-2-one			
	1-Phenyl-1-propanone			
**Nitriles**	4-Pentenenitrile	Italy	Fresh leaves HS-SPME	[19]
	3-Hydroxy-4-pentenenitrile			
	Heptanenitrile			
	Octanenitrile			
	2-Phenylacetonitrile			
	2-Pentenenitrile	Italy	Dried roots HS-SPME	[51]
	4-Pentenenitrile			
	2,4-Pentadiene nitrile			
**Fatty acids**	Palmitic acid	Turkey	Seed Chloroform-Methanol (2:1 v/v)	[52]
	Linoleic acid			
	Oleic acid			
	Linolenic acid			
	Stearic acid			
	11-Eicosenoic acid			
	Arachidic acid			
	Erucic acid			
	Behenic acid			
	15-Tetracosanoic acid			
	Tetracosanoic acid			
	Ursolic acid	Germany	Dried rosette leaves Dichloromethane	[33]
	Palmitoleic acid			
	α-Lysolecithin			
	(7Z, 10Z, 13Z)-Hexadecatrienoic acid			
	Corchorifatty acid B			
	9-Hydroxy-(10E, 12E, 14E)-octadecatrienoic acid			
	9-oxo-(10E, 12Z, 15Z)-Octadecatrienoic acid			
**Polysaccharides**			Root	[34]

**Table 2:** Biological activities of extracts and isolated compounds from *I. tinctoria* L.

Biological Activity	Experimental Model	Site of Collection	Plant Part/ Extract or Compound	Mode of Administration and Doses	Ref.
**Anti-inflammatory Activity**	Chronic *P. aeruginosa* lung infection mimicking cystic fibrosis in rat	Pharmac. factory of Sichuan Yaan	Aqueous	*s.c.* 400 mg/kg	[61]
	Micro-dialysis assay in the ex vivo pig foreleg skin	Germany	Dried rosette leaf Supercritical fluid Tryptanthrin	topical 0.5 g/10 mL + Try 0.115–1.84 mg/mL	[62]
	SLS-induced irritant contact dermatitis and UVB-induced erythema in healthy human volunteers	Germany	Dried rosette leaf Supercritical fluid Tryptanthrin	topical 50 µL 50 µL	[63]
	Carrageenan-induced paw oedema in mouse	Germany	Dried rosette leaf Dichloromethane Supercritical fluid Tryptanthrin	per os 75–125 mg/kg 125–175 mg/kg 70–40 mg/kg	[18]
	TPA-induced ear oedema in mouse	Germany	Dried rosette leaf Dichloromethane Supercritical fluid Tryptanthrin	per os 125 mg/kg 100 mg/kg 70 mg/kg	[18]
			Dried rosette leaf Dichloromethane Supercritical fluid Tryptanthrin	topical 0.5 mg/ear 0.5 mg/ear 0.25 mg/ear	
	TPA-induced ear oedema in mouse (sub-chronic inflammation)	Germany	Dried rosette leaf Dichloromethane	per os 150 mg/kg topical 1 mg/ear	[18]
	Delayed-type hypersensitivity induced by DNFB in mouse	Germany	Dried rosette leaf Dichloromethane	per os 150 mg/kg topical 1 mg/ear	[18]
	Adjuvant-induced arthritis in rats	Germany	Dried rosette leaf Dichloromethane	per os 150–250 mg/kg	[64]
	TNF-α and IL-1β production in RAW 264.7 macrophages	Germany	Dried rosette leaf Dichloromethane	25–100 µg/mL	[64]
	OVA-induced allergic airway disease (asthma) in mouse		Dried leaf Supercritical fluid	intranasal 10–100 μg/mouse	[65]
**Analgesic** **Activity**	Writhing test in mice	Germany	Dried rosette leaf Dichloromethane Tryptanthrin	per os 150–200 mg/kg 40 mg/kg	[18]
**Anti-tumour** **Activity**	Clinical trials (patients with chronic myelocytic and chronic granulocytic leukaemia)		Indirubin	per os 150–450 mg/day	[66]
	Human gastric cancer cells (HGC) Lung cancer cells (HLC) Promyelocytic leukaemia cells (HL-60)		Tryptanthrin	IC_50_ = 1.5 µg/mL 2.2 µg/mL 4.2 µg/mL	[67]
	Human monocytic (U-937) and promyelocytic (HL-60) leukaemia cells		Tryptanthrin	0.78–25 μg/mL IC_50_ = 3.1–6.3 μg/mL	[68]
	Azoxymethane-induced intestinal tumor in F344 rats		Tryptanthrin	per os 50 mg/kg	[69]
	Mammary carcinoma cell line (MCF-7) and Large cell lung tumour xenograft cell line (LXFL529L)		Indirubin	IC_50_ = 4.0 ± 2.0 μM 9.9 ± 0.1 μM	[70]
	MCF-7 cells and Doxorubicin-resistant breast cancer (MCF-7/adr) cells		Tryptanthrin	10^-6^ M	[71,72]
	Myelomonocytic leukaemia induced in BALB/c mice by WEHI-3B JCS cells		Tryptanthrin	i.p. 0.04–0.16 mg/kg/day	[73]
	Murine myeloid leukaemia (WEHI-3B JCS) cells		Tryptanthrin	0–5 µM IC_50_ = 1.5 µM	[73]
	Human chronic myeloid leukaemia K562 cells		Tryptanthrin	0.39–25 μg/mL IC_50_ = 8.8 μg/mL	[74]
	Xenograft human prostate tumour in BALB/c nude mouse model		Indirubin	Intralesionally injected 10 mg/kg/day	[75]
	Human umbilical vein endothelial cell (HUVEC) and Human prostate cancer cells (PC-3)		Indirubin	0–100 μM	[75]
	*N-myc* amplified human neuroblastoma LA-N-1 cells		Tryptanthrin	0–30 μM IC_50_ = 15.8 ± 1.41 μM	[76]
	Matrigel plug assay in BALB/c mice		Tryptanthrin	0–20 μM	[77]
	Human microvascular endothelial HMEC-1 cells		Tryptanthrin	0–20 μM	[77]
	Non-small cell lung cancer NCI-H460, human glioblastoma SF-268, and human breast cancer MCF-7 cells		Tryptanthrin	IC_50_ = 8.5 ± 0.8 μM 22.6 ± 1.1 μM 9.4 ± 0.3 μM	[78]
	Human anaplastic thyroid cancer cell lines CAL-62 and 8505C cells	Italy	Frozen and lyophilized cauline leaf Phenolic-rich fraction	0.01–0.1 mg/mL	[46]
	Human anaplastic thyroid cancer cell lines CAL-62, 8505C and C-643	Italy	Frozen and lyophilized Rosette leaf Cauline leaf Flower 70% Methanol	0.1–1 mg/mL	[47]
	DMBA/PMA-induced skin carcinogenesis model in *Swiss albino* mice		Tryptanthrin	topical 0.5–1 mg	[79]
**Antimicrobial activity**	Agar dilution test for bacteria, yeasts, and dermatophytes		Tryptanthrin	3.1–400 µg/mL	[80]
	Paper disc method for phytopathogenic microorganisms		Tryptanthrin	1–500 µg/mL	[80]
	Agar diffusion test for 23 micro-organisms. Sensitive strains: *Bacillus mycoides, B. subtilis,* tetracycline resistant *Micrococcus luteus*, and *Saccharomyces cerevisiae*	Germany	Fresh whole plant Water/Ethanol	‒	[81]
	Agar dilution test for synergistic activity with antibiotics against Methicillin-resistant (MRSA) and standard *Staphylococcus aureus*	China	Dried leaf 75% Ethanol	500 µg/mL	[82]
	Agar diffusion test for 15 micro-organisms. Sensitive strains: *Staphylococcus epidermis*, *S. aureus*, and MRSA		Tryptanthrin	12.5–100 µg/mL	[83]
	Microdilution broth method for MRSA		Tryptanthrin	15–1000 µg/mL	[84]
	Micro-titter plate method for 14 micro-organisms. Most sensitive strains: *B. subtilis, M. luteus* and *S. aureus*	Not indicated	Branches, flowers, leaves and roots Extracted with 14 different solvents	3.7–100 µg/mL for bacterial strains	[85]
**Antiviral** **activity**	Production of RANTES by Human bronchial epithelial cells H292 infected with influenza virus A/NWS/33 and B/Lee/40 – ELISA		Indirubin	100–200 μM	[86]
	Human influenza viruses (H1N1 and H3N2) and avian influenza viruses (H6N2 and H9N2) – MTT assay		Polysaccharides	IC_50_ = from 4.35 ± 0.07 to 28.20 ± 0.49 mg/mL	[87]
	Vero cells infected with Herpes simplex virus type II (HSV-2) - Cytopathic effect and MTT assay		Polysaccharides	25–800 mg/L	[88]
**Antioxidant** **activity**	1,1-diphenyl-2-picrylhydrazyl (DPPH) test		Leaf Hydroalcoholic	SC_50_ = 103.9 μg/mL	[89]
	2,2-Azino-bis-3-ethylbenzothiazoline—6-sulfonic Acid (ABTS) assay		Polysaccharides	Scavenging effect at 0.3 mg/mL= 64.3%	[34]
	1,1-diphenyl-2-picrylhydrazyl (DPPH) test		Dried *I. tinctoria* (plant part not specified) 95% Ethanol	IC_50_ = 1583.45 ± 23.69 mg/mL	[90]
	Trolox Equivalent Antioxidant Capacity (TEAC)			mM Trolox/g = 589 ± 0.51	[90]
	Reducing power assay			Abs_700_ = 0.32 ± 0.004	[90]
	1,1-diphenyl-2-picrylhydrazyl (DPPH) test		Indigo Indirubin	EC_50_ = > 0.26 mg/mL > 0.26 mg/mL	[91]
	Superoxide anion radical scavenging activity		Indigo Indirubin	EC_50_ = 0.61 mg/mL 0.74 mg/mL	[91]
	Hydroxyl radical scavenging activity		Indigo Indirubin	Not active Not active	[91]
	Reducing power		Indigo Indirubin	Not active Not active	[91]
	1,1-diphenyl-2-picrylhydrazyl (DPPH) test	Italy	Frozen and lyophilized cauline leaf Phenolic-rich fraction	IC_50_ = 0.6657 ± 0.0024 mg/mL	[46]
	Reducing power	Italy	Frozen and lyophilized cauline leaf Phenolic-rich fraction	ASE/mL = 3.87 ± 0.71	[46]
	Ferrous ions (Fe^2+^) chelating activities assay	Italy	Frozen and lyophilized cauline leaf Phenolic-rich fraction	Not active	[46]
	Protective effect on *Escherichia coli* under H_2_O_2_ stress	Italy	Frozen and lyophilized cauline leaf Phenolic-rich fraction	Not active	[46]
	1,1-diphenyl-2-picrylhydrazyl (DPPH) test	Italy	Frozen and lyophilized Rosette leaf Cauline leaf Flower 70% Methanol	IC_50_ = 1.151 ± 0.004 mg/mL 0.581 ± 0.001 mg/mL 0.437 ± 0.003 mg/mL	[47]
	Reducing power	Italy	Frozen and lyophilized Rosette leaf Cauline leaf Flower 70% Methanol	ASE/mL = 2.775 ± 0.163 1.546 ± 0.006 2.799 ± 0.042	[47]
	Ferrous ions (Fe^2+^) chelating activities assay	Italy	Frozen and lyophilized Rosette leaf Cauline leaf Flower 70% Methanol	IC_50_ = 1.234 ± 0.010 mg/mL 0.564 ± 0.011 mg/mL 0.856 ± 0.002 mg/mL	[47]
